# Recent Development of Nano-Carbon Material in Pharmaceutical Application: A Review

**DOI:** 10.3390/molecules27217578

**Published:** 2022-11-04

**Authors:** Prastika K. Jiwanti, Brasstira Y. Wardhana, Laurencia G. Sutanto, Diva Meisya Maulina Dewi, Ilmanda Zalzabhila Danistya Putri, Ilmi Nur Indira Savitri

**Affiliations:** 1 Nanotechnology Engineering, Faculty of Advanced Technology and Multidiscipline, Kampus C Universitas Airlangga, Surabaya 60115, Indonesia; 2 Department of Chemistry, Faculty of Science and Technology, Universitas Airlangga, Surabaya 60115, Indonesia

**Keywords:** carbon, multifunctional nanomaterial, sensor, drug delivery, good health and well-being

## Abstract

Carbon nanomaterials have attracted researchers in pharmaceutical applications due to their outstanding properties and flexible dimensional structures. Carbon nanomaterials (CNMs) have electrical properties, high thermal surface area, and high cellular internalization, making them suitable for drug and gene delivery, antioxidants, bioimaging, biosensing, and tissue engineering applications. There are various types of carbon nanomaterials including graphene, carbon nanotubes, fullerenes, nanodiamond, quantum dots and many more that have interesting applications in the future. The functionalization of the carbon nanomaterial surface could modify its chemical and physical properties, as well as improve drug loading capacity, biocompatibility, suppress immune response and have the ability to direct drug delivery to the targeted site. Carbon nanomaterials could also be fabricated into composites with proteins and drugs to reduce toxicity and increase effectiveness in the pharmaceutical field. Thus, carbon nanomaterials are very effective for applications in pharmaceutical or biomedical systems. This review will demonstrate the extraordinary properties of nanocarbon materials that can be used in pharmaceutical applications.

## 1. Introduction

Carbon is the fourth most abundant element in the universe. Carbon becomes the basis for all organic chemistry. Carbon and its derivatives have a main role in many high-performance materials, especially in electrochemistry. Various carbon-based materials have been studied and reported for numerous important applications [1,2]. As a green material, carbon has many advantages such as long stability, promoting stable bonding with many functional groups for material modification, low background current and wider potential window, which makes it widely applied in various fields, such as for synthesis [3,4,5], sensor [6,7,8], drug delivery [9,10], and therapy [11].

In recent years, development in nanotechnology has been tremendously improved. Nano carbon material has its own distinction for researchers around the world; carbon nanotubes (CNTs), fullerene, graphene, nanodiamond, are widely studied for numerous applications. Due to the excellent properties of carbon materials, their biocompatibility is very advantageous in medical and pharmaceutical applications. Several carbon materials such as carbon fiber [12], nanodiamond [13], carbon nanotubes (CNTs)/Chitosan [14], carbon dots [15], and diamond-like carbon [16], have been applied in bio-related applications including biomedical devices and bone implants due to their biocompatible properties.

Carbon nanostructured materials have a very large surface area that makes nanocarbon highly reactive compared to their bulk forms. The use of nanoparticle materials has high potential in sensor applications due to their outstanding electrical, chemical and mechanical properties. Some examples of carbon nanostructured materials that have been widely applied as sensors are CNTs, fullerenes, graphene and nanodiamonds. Since the last few years until now, many researchers have investigated nanocarbon-based sensors, especially their effectiveness and preparation methods. Due to their great physical and chemical properties, carbon nanomaterials have great potential in various biomedical aspects including bio-sensing, antibacterial action, cancer therapy, drug delivery and atherosclerosis treatment studies [17,18,19,20,21].

Recently, Porto et al., wrote a review on carbon nanomaterials: synthesis and applications to development of electrochemical sensors in the determination of drugs and compounds of clinical interest. The authors show that carbon nanomaterials have excellent thermal and electrical conductivity, strong adsorption capacity, high electrocatalytic effect, high biocompatibility, and high surface area. The possibility of forming different composite materials based on carbon nanomaterials to reduce the toxic effects of carbon nanomaterials makes this material promising for the development of analytical sensors, contributing to rapid, sensitive and low-cost analysis [22]. Additionally, Li et al., also reported that the development of carbon-based functional nanomaterials has the potential to be applied in various fields such as environmental science, pharmaceutical analysis and medical science [23]. Carbon nanomaterials have great potential to make significant future advances in analytical applications.

**Scheme 1:**
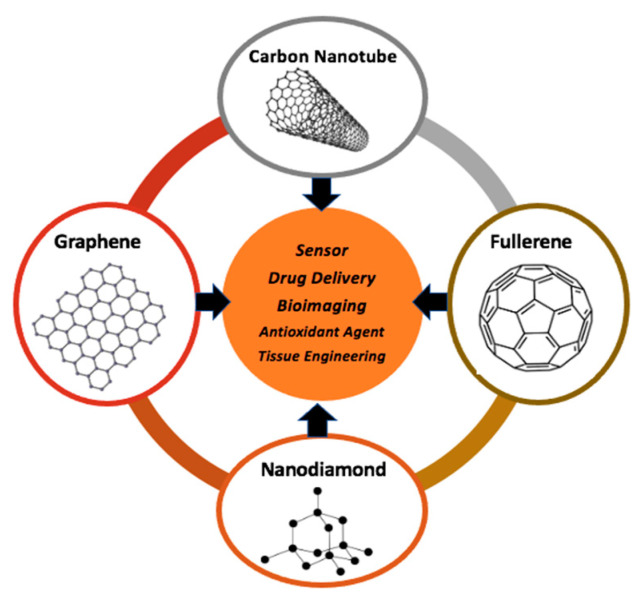
Schematic summary of nano-carbon material for various pharmaceutical applications.

In this review, the latest advances and developments in the use of carbon nanomaterials for pharmaceutical application will be presented. This review focuses on several types of carbon nanomaterials such as CNTs, fullerene, graphene and nanodiamonds for pharmaceutical applications, including sensor, drug delivery, antioxidant agent, tissue engineering and bioimaging application (Scheme 1).

## 2. Sensor Applications

The use of nanomaterials for the development of biosensors has attracted a lot of research interest. The unique structure of these nanomaterials allows them to interact non-covalently with organic molecules through various forces such as -π-stacking, hydrogen bonding, hydrophobic interactions, Van der Waals and electrostatic forces [24]. These interactions and their hollow structure make them excellent candidates for analytical applications. Carbon nanomaterials used as electrodes show high electrocatalytic properties. Nano-biosensors are beneficial because of their high surface area and surface volume ratio. In addition, it is often used for sensor applications because of its great chemical stability, and high biocompatibility compared to other conventional sensor materials.

### 2.1. CNTs

CNTs are carbon materials that are highly sensitive and selective due to its electronic properties which could adsorbed molecules in their surface [25]. Especially in pharmaceutical application, CNT can be used as both an electrochemical and photochemical sensor. Nowadays, many researchers are concerned with this kind of application (Table 1). One of its applications of CNT is being an isoniazid (INZ) detection sensor. Meanwhile, tuberculosis (TB) is categorized as one of the top ten diseases that cause death. This could be prevented by consuming INZ as a widely antibiotic for anticipation and diagnosis of TB [26]. Overdose of this drug can trigger seizures, metabolic acidosis, comas, and even death [27]. Thus, detection of isoniazid in the human body is very important to prevent unintentional things from happening. One of which is the detection of INZ level by using sensors.

A research conducted by Santos et al., proposed electrochemical sensor WS_2_/CNTs/GCE. Electrochemical sensor exhibit simplicity in their operational, rapidity, and real-time detection [28]. This sensor prepared by added WS_2_/CNTs nanocomposite which had previously been synthesized by sonication, centrifugation, and mixing with 5 mL ethanol. Next, a 30 min sonication process is carried out to form a suspension which will later be drop-casted into a polished glassy carbon electrode (GCE) until the ethanol is evaporated using mild heat from a blower. By combining the catalytic sites of WS_2_ with CNT that have good electrical conductivity and large surface area, it could improve the electrochemical activity of the sensor. The electrochemical activity was tested by using cyclic voltammetry (CV) and differential pulse voltammetry (DPV). Ferreira et al., (2016) developed an FePc/f-MWCNT/GC-modified electrode by polishing the surface of GCE using alumina slurry before the electrode was modified [29]. Then, the electrode was modified by a suspension that consisted of 2.0 mg f-MWCNT, 1.0 mg FePc, and 500 μL of dimethyl sulfoxide (DMSO). The electrode dried for 30 min at 50 °C to form FePc/f-MWCNT composite at the electrode surface. This iron phthalocyanine FePc/MWCNTs as electrochemical sensor for detecting INZ have many eminences, such as wide linear range, excellent sensitivity, and low limit of detection (LOD) at physiological conditions. In other side, Veerakumar et al., proposed a Zn@S-FeNC/f-CNTs electrochemical sensor modified in GCE via conventional hydrothermal and followed by ultrasonication [26]. f-CNT could enhance electrocatalytic of Fe_2_O_3_ towards detected molecules. So, the modifying of f-CNT in Zn@S-FeNC produce high active surface area with great electron transfer properties so that enhance the electrocatalytic performance of the composite and effectively detected INZ in real sample.

On the other hand, paracetamol has become a hottest drug after the COVID-19 situation because it’s effectiveness to reduce fever as one of the most common symptoms in the early stages of COVID-19 [30]. Latest in 2022, Teglia et al., developed GCE modified by MWCNTs-COOH using a mixture of natural deep eutectic solvent (NADES) [31]. NADES can be an alternative way to support green chemistry by using green solvents in chemical synthesis of material, which are environmentally friendly and non-toxic. The manufacture of modified GCE started with polishing 1.0, 0.30, and 0.05 mm alumina slurries to bare GCE for 2 min/each. Then, GCE were modified by dropping 10 μL of MWCNTs–LGH–EG dispersion on the top of the surfaces followed by evaporation of the solvent by exposure to stove at 50 °C for 10 min. The modified GCE resulting high stability, reproducibility, increase electrochemical active site and area. Modified electrode also resulting a simple, fast, sensitive, and selective electrochemical sensor for quantification of paracetamol. In addition, this sensor could be applied as quality control of medicines in pharmaceutical, industry, and detection of environmental and emerging pollutants. In other side, Shalauddin et al., proposed modified screen-printed electrode (SPE) with NNC-PPY/SWCNTs composite. The dispersion of NNC-PPY composite was made using ultrasonication method. Then the dispersion dropped into cleaned SPE by drop casting method. This novel sensor categorized as facile, highly selective, and metal-free nanosensor. Also, it has extraordinary electrical conductivity which could show excellent analytical response to determine paracetamol and ciprofloxacin whether in pharmaceutical preparation, biological fluids, until water sample.

Several researchers constructed electrochemical sensor using glassy carbon electrode (GCE) for acetaminophen (AP), another name of paracetamol. In the work developed by Alam et al., in 2018 to determine AP level in pharmaceutical and organic micro-pollutants monitoring in water by modified GCE with MWCNT/β-Cyclodextrin (CD). The fabrication of this electrode started with polishing GCE surface on fine emery paper and chamois leather containing alumina powder (Al_2_O_3_) then the electrode rinsed with DI water. After being rinsed, the electrode sonicated in DI water and dried with compressed air. An aliquot of 6–14 μL pf physically/chemically modified MWCNT/β-CD solution was drop casted into GCE and dried in the air for 20 min at 80 °C in an oven. The combination of these two elements could enhance sensitivity of the sensor due to large effective surface area, strong redox capability, and increasing host-guest interaction capabilities [32]. In another publication published by Wu et al., create a high selectivity, stability, and reproducibility AP sensor by modified GCE with Pd-MWCNT [33]. This modified sensor increases the roughness of MWCNT after being decorated by palladium (Pd). This modification could increase the AP absorption sites, which can increase the response current. The synergism between Pd and MWCNT that categorized as conductive materials can gain the electrochemical behavior of the sensor. The manufacturer process of the sensor is by polishing the surface of GCE with alumina powder having a diameter of 0.3 μm and 0.05 μm until mirror-like surface with the help of ultrasonication process. After that, following by ultrasonication cleaning with ethanol and DDW, 5 μL Pd-MWCNT suspension was dispensed into the surface of GCE and dries in air. The Pd-MWCNT composites were dispersed in DMF and ultrasonication for about 30 min until form a black suspension. Another research in AP sensor was conducted by Wester et al., in 2020, they developed electrochemical sensor by using SPE modified with Nafion-SWCNT [34]. This sensor form in a test strip which highly portable and fast point of care for screening AP. Moreover, the reference electrode of this sensor containing silver (Ag) that have many advantages, such as excellent shelf life, long term stability, and short hydration time. This sensor can be applied in real sample like plasma and finger-prick whole blood. The preparation of this sensor by patterned SWCNT electrodes with a pulsed nanosecond laser in wavelength of 1064 nm. Finally, the A4 PET film was coated with 117 Nafion which was diluted to 2.5% by weight with ethanol using a slot die coater. As the strip form, it was cutted and covered with poly(tetrafluoroethylene) (PTFE) with 6 mm pre-punched holes. Recently, in 2021 Charithra et al., also develop electrochemical based sensor for AP determination. They use carbon paste electrode (CPE) as the electrode and modified with CA/POAMCNT. This modified sensor possesses a high surface-active area. Thus, could be a significant AP detection in tablets and blood serum samples. The manufacturer of through the electrochemical polymerization process of OA on the surface of BCNTPE using CV. After the electrochemical polymerization, the electrode was subjected to immobilization of 10 μL of CA for about 5 min at room temperature. After 5 min, the electrode was thoroughly rinsed with double-distilled water [35].

Another CNTs based sensor application is used to determine amlodipine (AM). Amlodipine is dihydropyridine derivative that act as a calcium-channel blocker [36]. Naiko et al., develop electrochemical sensor using carbon paste electrode (CPE) modified with AgNP/f-MWCNT/CuNP. The manufacturer of the f-MWCNT/Cu hybrid CPE is by homogenizing 10.5 mg carbon powder to form a paste and the paste filled with 2 mm polyethylene syringes pre-inserted Cu wire for external electric contact. The the surface of the electrode modified with AgNP by immersing in solution of 10 mM AgNO_3_ containing 10 mM KNO_3_. As the result, combination of CuNP, AgNP, and f-MWCNT gain the electrocatalytic properties of the electrode due to high aspect ratio, good conductivity, long term stability, easier and faster charge transfer at electrode surface. This sensor also highly selective and sensitive in blood plasma and various pharmaceutical. In other side, Kokab et al., and Attal et al., develop electrochemical based sensor using glassy carbon electrode (GCE) to detect AM in real sample (table, drinking water, sweat/saliva) and human serum. In Kokab et al., they modified GCE with COOH-CNT/Ag/NH_2_-CNT. The synergy between fCNT and AgNP could increase sensitivity for target analytes and faster charge transduction for the redox probe [37]. This electrode was made by rubbed nylon cushion with μ-Al_2_O_3_ slurry on GCE surface until shine and smooth. After this the prepared electrode rinsed by ddw, ethanol, and aq. HNO3. The last by chemically cleaned by multiple reversible cyclic voltammetry. From this, GCE electrode was active and modified with NH_2_-CNTs, COOH-CNTs through drop-casting, and Ag NPs dispersions through mixed and layer-by-layer (LBL) methods. Another modified electrode provides by Atta et al., in 2019 GC/CNT/ILC/RGO/CW electrochemical sensor. The synergetic effect among all element can generate electrocatalytic activity, good sensitivities, lower LOD, and better precision of the layered sensor. This sensor constructed via casting, mixing, electrochemical reduction, and electro-polymerization method.

Another ciprofloxacin detection method is by electrochemical sensor with different modified electrode. In 2019, Jalal et al., develop GCE modified by PEI@Fe_3_O_4_@CNTs nanocomposite with wide potential window, high sensitivity, great electrocatalytic properties, and easy preparation method [38]. All of this advantage due to the synergetic effect between CNTSs and magnetite nanoparticle. The manufacturer of the PEI@Fe_3_O_4_@CNTs/GCE sensor was carried out by modifying the bare GCE which has been polished with alumina slurry until become mirror like surface and sonicated it in 5 min. Pretreatment step to produce carboxylic acid group on GCE surface reach by adding H_2_SO_4_ solution by cycling in the potential window −0.3 until +1.5 v with the scan rate 100 Mv/s. PEI@Fe_3_O_4_@CNTs composite suspension was dropped on the pretreatment GCE surface and rinsed with DIW to remove unbound reagent. In comparison, research conducted by Jorge et al., proposed cyclodextrin (CD)-incorporated multi-walled carbon nanotubes (MWCNTs) on polyaniline (PANI) modified GCE. Incorporate β-CD in PANI/MWCNT sensor could raise a significant signal improvement due to the increase of ciprofloxacin concentration at the electrode surface [39]. The preparation of this modified electrochemical sensor by polished GCE to mirror like finish with alumina powder and by drop casted f-MWCNT dispersion into the GCE surface and dried in air at ambient temperature. This sensor can be used for screening pharmaceuticals in wastewater treatment.

Another pharmaceutical drug that has been detected by CNT based sensor is Ivabradine hydrochloride (IVB). IVB is a heart rate-lowering drug that works through the selective inhibition of the pacemaker current for the treatment of heart failure, heart-related chest pain, sinus rhythm, angina pectoris when beta-blockers have no response [40]. Consume IVB in excess of the dose per day (overdose) can lead to severe and prolonged bradycardia, uncontrolled blood pressure, headache, and blurred vision [41]. Because of this problem, there is analytical technique for IVB determination, one of it by potentiometric sensor. Potentiometric has several advantages, for instance wide linear dynamic range, sub-micromolar detection range, absence of an inner filling solution, fast response to analyte, and reliable selectivity [42]. The electrode of this potentiometric sensor is using carbon paste electrode (CPE) modified with Fe_2_O_3_@MWCNTs by inserted the paste packed into the electrode body hole then burnished onto a smooth paper until a shiny appearance exists. The combination between CPE and Fe_2_O_3_@MWCNTs can increase active site and rate of mass transport to the electrode surface. Thus, could increase slope and linear dynamic range, and decrease detection limit. From this sensor, the author determine that this sensor is simple with high selectivity, short response time, and feasible. In addition, Fe_2_O_3_@MWCNT can be utilized for IVB determination not only in pharmaceutical formulations but also in physiological fluids (e.g., plasma, urine, and serum).

**Table 1:** Summary of CNTs for pharmaceutical sensor application.

Electrode	Modifier	Detection Method	Target	Sample	LOD (μM)	Linear Range (μM)	Electrolyte	pH	% Recovery	Ref.
GCE	WS_2_/CNTs	CV, DPV	Isoniazid	Urine	0.24	10.0–80.0	KCl, HCLO_4_ (only for hydroquinone)	7	96.9–104.5	[43]
	FePc/f-MWCNT	CV		Saliva, Blood	0.56	5–476	NA.	7.4	97.3–104.0	[29]
	Zn@S-FeNC/f-CNT	CV, DPV		Human Blood Serum, Urine	0.00501	0.05–230.5	PBS	7	96.12–99.1	[26]
		Amperomety			0.00854	0.07–233.4				
GCE	MWCNTs–LGH–EG	CV, LSV, DPV	N-(4-hydroxyphenyl) acetamide (paracetamol)	Urine	0.1	0.100–7.510	PBS, BBS	10	90–92	[31]
SPE	NNC-PPY/SWCNTs	CV. LSV, EIS	Paracetamol (PCM)	Lake Water	0.000072	0.05–40.0	PBS	7	98–105	[44]
			Ciprofloxacin		0.000196	1.00–50.00	PBS	7	99–102	
GCE	PANI–β–CD/fMWCNTs	CV		Water sample	0.05	10.00–80.00	PBS	6	98.2–107.0	[39]
	PEI/FE_3_O_4_/CNTs	DPV		Drug tablets, urine, serum	0.003	0.03–70.00	B-R Buffer (Britton Robinson), KCl	6.5	97–108	[38]
	COOH-CNTs/Ag/NH_2_-CNTs)	CV	Amlodipine	Tablets, tap/drinking water, sweat/saliva, urine/serum sample	7.76 × 10^−8^	6 nM–50 pM	PBS	6	95–102	[37]
	GC/MWCNT/ILC/RGO/CW	CV		Human serum	0.000139	0.008–30	PBS	7.4	99.77	[36]
Carbon Paste Electrode (CPE)	AgNPs/fMWCNT/Cu-NPs	CV, EIS, AdSWV		Drug tablets, urine, serum, plasma	0.000516	0.02–6.3	Britton-Robinson (BR) buffer	10.5	99.00–100.75	[45]
	POA/CA	CV, EIS, DPV	Acetaminophen	Blood serum, tablet	0.015	2.0–10.0 & 15.0–50.0	PBS	7	98–101	[35]
GCE	GC/MWCNT/ILC/RGO/CW	CV		Human serum	0.0000906	0.001–20	PBS	7.4	98.96	[36]
	MWCNTs/β-cyclodextrin (β-CD).	CV, LSV		Drinking Water, Urine	0.0033	0.005–20	PBS	7.4	98–101	[32]
	Pd-MWCNT	CV		Real sample	0.13	0.5–100	PBS	7	96.0–101.1	[33]
SPE	Nafion-SWCNT	CV, DPV		Plasma	0.8	1.00–2000	PBS	7.4	79	[34]
				finger-prick whole blood					74	

### 2.2. Fullerene

In 2013, Rather & De Wael [46] developed an electrochemical sensor fabricated by modifying GCE using 98% pure C_60_ for Bisphenol A (BPA) detection. BPA is an estrogenic toxin that is widely used in the manufacture of plastics that has the potential to harm human health with various side effects such as impaired brain development, sexual differentiation, and immune function. [47,48,49]. The sensor is made by the electrodemodification method. C_60_ stock solution prepared from 150 M CH_2_Cl_2_ solution was adsorbed onto the polished surface of the GCE and rinsed until it resembled a mirror. The C_60_ film formed was reduced in 1.0 mol/L KOH at a potential of 0.0–1.5 V at 10 mV/s then the electrode was equilibrated into phosphate buffer pH 8.0 by cyclic scanning at a potential of 550 mV–50 mV at 20 mV/s for 20 min at under a nitrogen atmosphere. The assembled sensor is stored at +4 °C. The fabricated C_60_/GCE sensor exhibited excellent electrocatalytic activity in lowering the anodic over-potential and a remarkable increase in the anodic current of BPA. This sensor has easy detection steps and is unaffected by the presence of various other endocrine disruptors, is simple to manufacture, and has good reproducibility. Although the addition of C_60_ to GCE improved its performance, the addition of too much C_60_ (>30 µL) had the potential to reduce the peak oxidation current due to saturation of the electrode surface.

In 2014, Mazloum-Ardakani & Khoshroo [50] fabricated electrochemical sensors made of C_60_-functionalized carbon nanotubes (CNTs)/ionic liquid (IL) composites modified GCE to detect several catecholamines, namely norepinephrine (NE), isoprenaline (IP), and dopamine (DA). Catecholamines are a group of hormones that have catechol groups that are normally released in response to physical or emotional stress. The modification begins with GCE polishing on the polishing cloth using 0.05 m alumina powder. MWCNTs/C_60_ with a mass ratio of 2:1 and a total weight of 1 mg were dispersed in 10 mL toluene in an ultrasound bath for 30 min to make a suspension of 0.1 mg/mL. Next, 10 µL of the suspension was applied directly to the surface of the GCE and allowed to stand at room temperature for 10 min to evaporate the solvent. After that, the electrode was scanned in ACN containing 0.1 M tetrabutylammonium hexafluorophosphate with a potential range of 0.0–2.0 V to obtain a reversible multistep electron-transfer reaction. The resulting electrode was then washed with ACN several times to remove the electrolyte and then dried at room temperature. Furthermore, a mixture of 50 µL 1-butyl-3-methylimidazolium tetrafluoroborate (IL) which had been dispersed into 0.3 mL of 1% chitosan (CH) solution in 1.0 M acetic acid was sonicated for 30 min and applied to the electrode surface as much as 3 µL using a microsyringe and then dried. in hot air for 15 min. The resulting C_60_-CNTs/IL/GCE sensor can sensitively detect catecholamines by a simple method. The presence of C_60_-CNTs/IL was also shown to increase the electrocatalytic activity of catecholamines. From the CV analysis, a pair of redox peaks from the sensor are well defined and do not change after 50 cycles indicating good stability and reproducibility of the sensor. Although the sensor performance is mostly supported by C=, the modification of C60 mostly changes the porosity or substrate accessibility of the electrode surface.

In 2015, Mazloum-Ardakani et al. [51] re-developed an electrochemical sensor fabricated from C60-functionalized CNTs/IL nanocomposite for the simultaneous determination of hydrazine and hydroxylamine. Hydrazine and hydroxylamine are widely used in the chemical industry and as raw materials for the synthesis of pharmaceutical substances, but these two compounds have toxic effects on humans, animals, and even plants [52,53]. This sensor is made by a simple casting modification method as in the previous paper [50]. Before being modified, GCE was polished first. MWCNTs/C_60_ were made by making a suspension of 0.1 mg/mL and as much as 10 µL of the suspension was dripped directly on the surface of the GCE. The electrodes were scanned in an ACN solution containing 0.1 M tetrabutylammonium hexafluorophosphate at a potential of 0.0–2.0 V to obtain a reversible multistep electron-transfer reaction. The resulting electrode was washed with ACN several times to remove the electrolyte and then dried at room temperature. 50 µL of IL was dispersed in 0.5 mL of 1% CH solution in 1.0 M acetic acid and sonicated for 30 min. 3 µL of the dispersion was dripped onto the surface of the GCE using a microsyringe and then dried in hot air. The prepared C_60_-CNTs/IL/GCE sensor exhibits a sensitive and simple determination of hydrazine and hydroxylamine with a low detection limit so that it can be used to determine hydrazine and hydroxylamine in real samples. Determination of hydrazine and hydroxylamine can run simultaneously without any interference effect. This sensor is at risk of decreased sensitivity if there is the formation of N_2_ and N_2_O gases from the oxidation of hydrazine and hydroxylamine. In the same year, Palanisamy et al. [54] synthesized an analytical electrochemical sensor made from palladium nanoparticles (PdNPs) decorated activated C_60_ (AC_60_) modified screen printed carbon electrode (SPCE) to detect AD. DA is a hormone that plays an important role in the human central nervous system which is very important to pay attention to because it is associated with several neurological diseases, such as Parkinson’s disease [55,56]. The sensor was made by dispersing 0.5 mg/mL of C_60_ in toluene and then sonicated for 20 min. The dispersion of 8 L was used to modify the SPCE by drop casting method. The modified SPCE was activated in 1 M KOH solution with a scanning potential of 0.0–1.5 V at a scanning rate of 0.01 V/s for two cycles then dried at room temperature and transferred to an electrochemical cell containing 0.5 mM PdCl_2_ in 0.5 M H_2_SO_4_. As much as 10 consecutives cyclic voltammograms were performed at a potential of 0.25–1.2 V at a scan rate of 50 mV/s to precipitate PdNP on the SPCE. The prepared AC_60_/PdNPs/SPCE sensor showed high sensitivity and selectivity in detecting DA with lower oxidation potential than SPCE modified by other materials. This sensor also has good stability and reproducibility and can detect AD practically so that it has the potential to be used for the detection of AD in pharmaceutical samples. Although this sensor has many advantages, the sensitivity and potential of this sensor is highly dependent on optimization.

In 2016, Thirumalraj et al. [57] also synthesize electrochemical sensors to detect DA. DA is a catecholamine from the phenethylamine family that helps regulate movement and the human body’s emotional response [58]. DA deficiency can cause several health problems such as Parkinson’s disease, attention deficit hyperactivity disorder (ADHD), and restless legs syndrome (RLS) [59]. This sensor is made from modified GCE using graphene oxide (GO) and C60 nanocomposite. GO was made from natural graphite (diameter < 20 µm) by the modified Hummers method and then purified with HCl and water and then dried at room temperature. Furthermore, GO was dispersed in water as much as 2 mg/mL with ultrasonication for 30 min to make GO solution. The GO-C_60_ nanocomposite was synthesized by adding C60 with a purity of 99.5% to the GO solution in a ratio of 1:2 and then sonicated at 45 °C for 6 h until the GO color changed to dark brown. C_60_ was dispersed in toluene as much as 1 mg/mL and sonicated to make a solution of C_60_. The prepared GO-C_60_ nanocomposite was centrifuged to remove unattached C_60_ and GO and then redispersed into water for further experiments. The GCE to be modified was first polished with alumina slurry and sonicated in a mixture of ethanol and water for 2 min. Approximately 6 µL of GO-C_60_ nanocomposite dispersion was dropped onto GCE and then dried at room temperature. The manufactured GO-C_60_/GCE sensor exhibits higher sensitivity, good selectivity and reproducibility, and lower overload potential to detect DA than bare GCE. The sensor also exhibits a more extensive and practical linear response in detecting AD in rat brain and commercial DA injection samples making it possible to use it to detect AD in pharmaceutical samples. However, this sensor is still not very selective if the concentration of interfering substances such as uric acid, glucose, ascorbic acid, etc. is too high. In the same year, Brahman et al. [60] fabricated electrochemical sensors to detect paracetamol (PT). PT is an effective analgesic and antipyretic drug and is often used to relieve pain [61]. PT has no toxic effect on human health when consumed in normal doses, but overdose of PT can lead to accumulation of toxic metabolites that can cause serious illness [62]. This sensor is made by modifying the carbon paste electrode (CPE) with copper nanoparticles (CuNPs)/C_60_/MWCNTs composite film modified. Before being modified, CPE was made by mixing graphite powder and paraffin oil in a *w*/*w* ratio of 70:30 to produce a homogeneous paste. After that, some of the paste is put into the bottom of the polyethylene syringe and smoothed on a weighing paper. Subsequently, the electrode was ultrasonicated in distilled water for about 30 s and then dried at room temperature. CuNPs/C_60_/MWCNTs composite films were prepared by mixing MWCNT and C_60_ in a ratio of 2:1, then 1 mg was dispersed into 10 mL toluene and ultrasonicated for 30 min to make 0.1 mg/mL suspension. 25 µL of the suspension was cast directly on CPE and dried at room temperature. The modified CPE was then immersed in a solution of 10 mM CuSO4 in 0.05 M H_2_SO_4_ and deposited for 20 cycles in a potential range of −0.3–1.0 V at a rate of 50 mV/s. The deposition time is determined in the range of 10–150 s, but the deposition time exceeding 100 s causes oxidized PT to cover the entire surface of the CPE which has the potential to degrade sensor performance. After that, the CPE was rinsed with double-distilled water and then dried at room temperature. The fabricated CuNPs/C_60_/MWCNTs/CPE sensors exhibit excellent selectivity and sensitivity in detecting PT with very low detection limits, wide linear dynamic range, and good reproducibility and repeatability.

In addition, there are studies by Mazloum-Ardakani et al. [63] which manufactures electrochemical sensors for the simultaneous sensitive determination of levodopa (L-dopa) and AP. L-dopa is widely used as a source of dopamine for the treatment of Parkinson’s disease and epilepsy [64,65]. People with Parkinson’s disease generally use pain relievers such as AP. Long-term use of L-dopa and AP can have a toxic effect on the body, so it is important to control their concentration in the body [66]. This sensor is made from a C_60_-functionalized CNT composite with a sensor manufacturing method similar to [50,51]. The composite was prepared by dispersing MWCNT and C_60_ in a 2:1 ratio and a total weight of about 1 mg into 10 mL toluene in an ultrasound bath for 30 min to make 0.1 mg/mL suspension. The GCE to be modified is polished with alumina powder and rinsed thoroughly with water and then dried. The dried GCE was dripped with 15 µL of suspension then dried and scanned in ACN solution containing 0.1 M tetrabutylammonium hexafluophosphate as a support electrolyte at a potential of 0.0–2.0 V to obtain a reversible multistep electron-transfer reaction. The modified electrode was washed several times with ACN to remove the electrolyte and then dried in hot air. The resulting C_60_-CNT/GCE sensor can determine L-dopa and AP simultaneously in real samples with a fast, sensitive, selective, and low detection limit so that it can be used for routine analysis. Although this sensor exhibits high electrocatalytic activity for the oxidation of L-dopa and AP, the sensitivity of this sensor can decrease when the mass of L-dopa and AC in the sample exceeds the detection capability of the sensor.

In 2017, Rahimi-Nasrabadi et al. [67] developed an electrochemical sensor for detecting diazepam. Diazepam is a benzodiazepine family drug that is commonly used to treat anxiety, antidepressants, sleep disorders, and seizures. Short-term use of diazepam is safe but long-term use of diazepam can cause dependence and other side effects, even diazepam overdose can cause death [68]. The manufacture of the sensor begins with GCE polishing which will be modified using alumina powder and washed with double-distilled water for 10 min. To make C_60_-CNT/IL nanocomposite, 5 mg of purified MWCNT and C_60_ in a ratio of 2:1 were dissolved in 10 mL of toluene using an ultrasonic bath then filtered and rinsed with ACN and then dried at 40 °C under vacuum overnight. After that, 2 mg of C_60_-CNT was evenly dispersed in 4 mL of ethanol and followed by the addition of 30 µL of 1-butyl-3-methylimidazolium tetrafluoroborate and then ultrasonicated. The built-in C60-CNT/IL/GCE sensor can detect diazepam in tablet, urine, and serum samples sensitively within low detection limits making it possible to use it in routine analytical control of drugs. This sensor also significantly increases the current reduction of diazepam and positively shifts the peak reduction potential of diazepam. Although this sensor is sensitive in detecting diazepam, it is still less selective if the foreign substance concentration exceeds 0.1 M.

In 2020, Anusha et al. [69] fabricated an electrochemical sensor of C_60_ and a bimetallic nanoparticle composite film to detect vitamin D3 in blood samples. Vitamin D3 is important for maintaining bone health and preventing some diseases such as Rickets [70,71]. Vitamin D3 deficiency can increase the risk of developing severe disease. The manufacture of this sensor begins with modifying the GCE with C_60_. 1 mg/mL standard solution C60 was prepared in toluene using a sonicator and then 5 µL of the resulting suspension was printed on GCE and dried under an infrared lamp for 30 min. Electrochemical reduction of the electrode was carried out in 1.0 M KOH using 10 cycles with a potential between −1.0–0.01 V at a rate of 10 mV/s then the electrode was transferred into a cell containing 0.5 M H_2_SO_4_ and 10 mM CuSO_4_. CuNPs were electrodeposited in a potential between −0.3–1.0 V at a rate of 50 mV/s. CuNPs@reduced-C_60_/GCE was immersed in a 10 mM nickel sulfate solution containing 0.4 M tri-sodium citrate and deposited in a potential between 0.1–1.6 V at a rate of 30 mV/s. The fabricated NiNPs-CuNPs@reduced-C_60_/GCE sensor showed increased sensitivity in detecting D3 when compared to conventional methods and other electrochemical sensors. The sensor also exhibits a wide linear range, low detection limit, good reproducibility, and long term stability. Although attention to the optimum composition of ethanol for dissolving D3 should be increased because it can interfere with the peak current value, the selectivity of this sensor is still acceptable. In the same year, Zhu et al. [72] synthesized electrochemical sensors based on C_60_ composites and platinum nanoparticles (PtNPs) for the selective determination of catechol (CC) and hydroquinone (HQ). CC and HQ are contaminants resulting from many industrial procedures that have high toxicity to the environment and humans even at very low concentrations. [73,74,75]. The manufacture of this sensor begins with the manufacture of a Pt/C_60_ composite from a mixture of 1 mL of C60 solution and 2 mL of PtNPs which is sonicated for 12 h in an iced water bath. Modification of the composite on the electrode was carried out by dripping 8 µL of the Pt/C_60_ mixture on the cleaned surface of the pyrolytic graphite electrode (PGE). The fabricated Pt/C_60_/PGE sensor exhibits high electrocatalytic activity from the synergistic effect of PtNPs and C_60_. The determination of CC and HQ can be carried out sensitively and selectively with a low determination limit and a wide linear concentration range. In addition, this sensor can produce large peak separations to counteract CC and HQ oxidation. This sensor has long term stability and good reproducibility despite easy and simple fabrication. Apart from the easy and simple fabrication process, optimization of the number of modifiers must be considered because it can affect sensor performance.

Furthermore, there is an electrochemical sensor for caffeine determination (CAF) developed by Taeju et al. [76]. Caffeine is one of the most widely consumed substances because of the drowsiness it produces. However, caffeine in high concentrations is toxic to the body and can cause nausea, shaking, hyperactivity, nervousness, and even death when consumed at concentrations above 200 mg/day. [77,78,79,80,81]. This sensor is made by drop-coating method. Prior to modification, GCE was polished with alumina paste then placed in a 1:1 ethanol-water solution and cleaned in a sonicator for 10 min to dissolve the remaining alumina. C60/MWCNT/Nafion dispersion was prepared by dispersing 3 mg C_60_/MWCNT into 1 mL Nafion 1% and then ultrasonic. GCE surfaces were coated with 2 µL of each dispersion and dried at room temperature for 15 min. The C_60_/MWCNT/Nafion/GCE sensor can analyze CAF quickly and sensitively making it suitable for routine determination of CAF in real samples. Although the selectivity of this sensor is not very high, the preparation of this sensor is easy and simple and has good stability and reproducibility so that it can be used for a long time. In 2021, Abdellatef et al. [82] synthesized electrochemical sensors for voltametric determination of rifaximin. Rifaximin (RFX) is one of the most commonly used antimicrobial agents in treating various infectious diseases, so rapid, sensitive, and selective control of RFX is needed. This sensor is based on manganese dioxide (MnO_2_) and C_60_ nanocomposites. The nanocomposite was prepared by mixing aqueous solutions of potassium permanganate (KMnO_4_) with manganese (II) sulfate (MnSO_4_) and adding concentrated nitric acid to an acidic pH of 1.0 and then curing at 80 °C for 4 h. Subsequently, the reaction products were collected by centrifugation and washed several times and then dried at 25 °C. CPE was prepared by mixing 1 g of graphite powder and 250 µL of paraffin oil in a ceramic mortar for 15 min and then packed into individual Teflon piston holders with electrically conductive screws. The resulting CPE was modified by the drop-casting method of 10 L nanocomposite suspension 3 times onto the CPE and then dried at 25 °C. The modified CPE was rinsed with double-distilled water and then put into an electrochemical cell. The resulting MnO_2_/C_60_/CPE sensor can detect RFX in real samples and pharmaceutical formulations quickly and sensitively. Modification of the nanocomposite on the sensor resulted in an increase in electrocatalytic activity against RFX oxidation. The degree of selectivity of this sensor is still unknown as no tests have been conducted. Moreover, in the same year there was a study by Materón et al. [83] who developed an electrochemical sensor for the sensitive detection of metronidazole (MTZ). MTZ is a synthetic antibiotic to treat trichomoniasis, dysentery, liver abscess, rosacea and burns due to anaerobic infections, and for surgical prophylaxis [84,85]. MTZ detection also aims to minimize its side effects, such as nausea, diarrhea, neurotoxicity, optic neuropathy, peripheral neuropathy, and antepatopathy. The sensor is made of SPE coated with C_60_, reduced graphene oxide (rGO) and Nafion. SPE is placed in a bath containing 0.5 mol/L H_2_SO_4_ while stirring for 1 min. 2 mL suspension containing 3 mg rGO and 50 µL Nafion 50% *v/v* was prepared and ultrasonicated for 20 min to make a homogeneous dispersion. Aliquots of 3 µL of C_60_ solution prepared in CH_2_Cl_2_ were printed onto the SPE surface and dried for 1 h. The electrodes were scanned by cyclic voltammetry for 2 cycles in 1 mol/L KOH solution with a potential of −1.5–0.0 V at a rate of 20 mV/s. Another scan was carried out in a phosphate buffer solution (PBS) pH 7.0 with a potential of 0.05–0.55 V at a rate of 50 mV/s. The prepared C_60_-rGO-Nafion/SPE sensor showed good electrocatalytic activity in detecting MTZ. This sensor has high stability, good repeatability and reproducibility, fast response, and low cost. These sensors hold promise for drug monitoring against bacterial resistance. The the utilization of fullerene modified on carbon-based electrode for pharmaceutical sensor application is summarized in Table 2.

### 2.3. Graphene

In 2012, Xi & Ming [86] developed a sensor for the sensitive determination of midecamycin (MD). MD is a macrolide antibiotic that has high antibacterial activity against Gram-positive and Gram-negative bacteria and is widely used for the treatment of upper and lower respiratory tract infections, acute laryngopharyngitis, tonsillitis, pneumonia, otitis media, urinary tract infections, and tissue infections. soft skin. The development of this sensor aims to determine the optimal concentration of MD in therapy and avoid MD overdose which can be dangerous. The manufacture of the GO/GCE sensor was carried out by modifying the GCE which had been polished with alumina slurry until the surface was like a mirror and sonicated for 3 min. GO was synthesized from spectral graphite using the modified Hummers method and then dialyzed for one week to remove acid and salt residues. GO that had been dried overnight at 50 °C was ultrasonicated to make 1 mg/mL of stable and homogeneous GO dispersion. 10 µL of GO dispersion was dripped onto the surface of the GCE and allowed to evaporate at room temperature. The modified GCE was immersed in 10 mmol/L PBS pH 5.0 and then reduced electrochemically at a potential of 1.5 V for 10 min. This sensor is sensitive and selective for the determination of MD in pharmaceutical formulations and biological fluids, and has good long-term stability. The sensor also exhibits high conductivity and excellent electrocatalytic activity derived from GO, however the excellent electrocatalytic activity of GO can cause adsorption of MD or its oxidation products to the electrode surface resulting in inactivation of the electrode surface.

In 2013, Arvand & Ghodsi [87] fabricated a graphene-modified sensor (G) for the determination of the amount of L-dopa in rat brain extracts and drugs. L-dopa is a drug that is widely used as a source of dopamine in the treatment of Parkinson’s disease and epilepsy [88]. The amount of L-dopa in the body needs to be monitored because it has serious side effects when used long term [89]. The G/GCE sensor was prepared by synthesizing G sheets by chemical reduction of graphite oxide and then dissolved in 10 mL of 10 mg N,N’-dimethylformamide (DMF) with ultrasonic assistance for 1 h to make a uniform black solution. GCE which has been polished with alumina powder to a mirror like and washed with double-distilled water and 1:1 ethanol is dried at room temperature before being modified. 5 µL of G-DMF suspension was poured on the surface of the GCE and the electrodes were dried at room temperature. G/GCE was rinsed with double-distilled water to remove G adhering to the electrode surface. This sensor has a broad specific surface which results in increased detection capacity and reversibility of L-dopa in pharmaceutical samples and mouse brain extracts. This sensor has good repeatability despite its simple, fast and inexpensive fabrication. The performance of this sensor is highly dependent on pH, when the pH is above 6.7, the L-dopa is reversible. The sensor also becomes saturated with L-dopa after an increase in accumulation time of up to 240 s.

In the same year, Arvand & Gholizadeh [90] also developed sensors for the determination of indomethacin. Indomethacin is a non-steroidal anti-inflammatory agent that is widely used in the treatment of inflammatory and degenerative diseases [91,92]. Gold nanorods (GNRs) were prepared in a seed solution containing 0.25 mM HAuCl_4_ and 0.25 mM tri-sodium citrate. The seed solution was added with 0.1 M NaBH_4_ cold solution while stirring until the color changed to orange-red indicating the formation of gold nanoparticles (AuNPs). GO was prepared from a mixture of 1 g of pure graphite powder and 8 g of potassium chlorate in 20 mL of fuming nitric acid at room temperature. The GO synthesis was followed by a washing, filtering, and cleaning process using the Brodie method. The prepared 10 mg GO was sonicated for 1 h and centrifuged at 15,000 rpm for 10 min to extract GO sheets. Pure MWCNTs were purified by reflux in nitric acid and then washed with double-distilled water to pH 7 and dried at 40 °C for 3 h. GNRs-GO nanocomposites were prepared by dispersing 10 mg of GO in distilled deionized water by sonication and then 10 mL of GO suspension was sonicated with 3 mL of GNR dispersed in deionized water at 25 °C for 1 h. 4 mg of the centrifuged and dried GNR-GO nanocomposite was mixed with a CNT paste (CNTP) containing 20 mg MWCNTs and 8 mg paraffin. The synthesized GNRs-GO-CNTP were used to modify GCE. This sensor produces high electrochemical activity and response when used for the detection of indomethacin. Modified GNRs-GO on CNTP also showed improved stability and good reproducibility, and had good selectivity and antifouling ability. Upon detection of indomethacin, the oxidation potential shifted to a less positive value as the pH of the medium gradually increased.

In 2014, Beitollahi et al. [93] synthesize sensors for determination of methyldopa in urine and pharmaceutical formulations. Methyldopa is an antihypertensive drug for treating high blood pressure, especially when it is accompanied by complications of kidney disease. The sensor is made from a graphene paste electrode (GPE) modified 2,7-bis(ferrocenyl ethyl) fluorene-9-one (2.7 BF). The 2.7 BFGPE electrode was prepared by mixing 0.01 g 2.7-BF with 0.89 g graphite powder and 0.1 g graphene nanosheet (GNS) which had been prepared previously from natural graphite by the Hummers and Offeman method in a mortar. The mixture was added about 0.7 mL of paraffin and stirred for 20 min until an even base paste was obtained. Pasta is packaged in the end of a glass tube i.d. 3.4 mm long and 10 cm long. Copper wire is inserted into the paste to provide electrical contact. This sensor exhibits an increase in sensitivity that comes from an increase in the electrode area. The electrode performance was improved in the presence of a modifier which resulted in a higher anodic peak current for methyldopa oxidation and electrocatalytic activity for methyldopa redox. Although this sensor has a wide linear range and low detection limit, it has a limited range of pH conditions, limited electron transfer kinetics, and is not very selective. In the same year, Rosy et al. [94] fabricates sensors for electrochemical analysis of NE in pharmaceuticals and biological fluids. NE is a catecholamine that has physiological side effects, such as anxiety, diabetes, pain, heart disease, and some neurological disorders such as Parkinson’s and Alzheimer’s disease [95,96,97,98,99]. The palladium (Pd) electrode was polished using alumina and zinc oxide on a microcloth pad before being modified with graphene. Graphene was synthesized from reduced GO by adding 20 mL of water to 50 mg of GO powder and then adding 0.5 mL of hydrazine. The mixture was sonicated for 1 h then continued with stirring for 24 h at 50 °C then filtered and dried in vacuum. Suspension graphene was prepared by dispersing 7 mg graphene in a mixture of double distilled water and 1:9 DMF at a concentration of 0.7 mg/mL. 7 µL graphene was cast on the surface of the Pd electrode and dried at room temperature. Modification of graphene on the Pd electrode resulted in a 2-fold increase in surface area and a 20-fold increase in sensitivity. This sensor can detect NE quickly even within low detection limits. However, this sensor is very dependent on pH, when the pH increases, the NE oxidation potential will shift towards a less positive value, besides this electrode reaction is controlled by a diffusion mechanism.

In 2015, Shrivastava et al. [100] synthesized sensors for the electrochemical detection of aripiprazole (ARP) in pharmaceutical formulations. ARP is an atypical antipsychotic and anti-depressant widely used in the treatment of schizophrenia, bipolar disorder, major depressive disorder and autism-related irritability [101,102,103]. The sensor is made of GCE modified graphene nanoparticles, titanium dioxide nanoparticles (TiO_2_ NPs), and polyaniline (PANI). PANI nanofibers were synthesized by interfacial polymerization technique using ammonium persulfate as oxidizing agent. graphene/TiO_2_/PANI nanocomposites were synthesized by dispersing graphene nanoparticles, TiO_2_NPs, and PANI in DMF in a ratio of 2:1:1 and then sonicated for 6 h to obtain a suspension of 1 mg/mL. GCE was polished with an alumina slurry on a Buehler cloth and rinsed periodically with ionized water before modification. 5 µL of graphene/TiO_2_/PANI nanocomposite was dripped onto the surface of the GCE and then dried at room temperature. Electrode performance is increasing with the presence of graphene/TiO_2_/PANI nanocomposite which results in higher sensitivity, specificity, reproducibility, stability, and accuracy. The voltammogram characteristics of this sensor depend on the pH of the medium, the peak becomes more unstable when the pH is above 3.8 and disappears completely, there is even some precipitate formation in the electrochemical cell when the pH is above 5.6. In the same year, Radhakrishnan et al. [104] synthesize sensors for sensitive determination of HQ. HQ sensors have proven useful for a wide range of applications, particularly the pharmaceutical and cosmetic industries, due to their toxic nature to humans and animals and difficult to degrade ecologically [105,106]. This sensor is made of GCE modified PANI-Fe_2_O_3_-rGO composite. GCE was polished using an alumina suspension then sonicated for 10 min and rinsed with water before being modified. Fe_2_O_3_-rGO composites were prepared by dispersing 50 mg GO into 70 mL ethanol and ultrasonic for 30 min and then adding 0.1 g of FeCl_2_ and 0.1 mL of 25% ammonia. The mixture was stored in a steel autoclave at 170 °C for 4 h and then cooled to room temperature. The precipitation was filtered and then washed with distilled water and ethanol several times and then dried at 80 °C for 6 h in a vacuum oven. The PANI-Fe_2_O_3_-rGO composite was prepared by adding the Fe_2_O_3_-rGO composite to 45 mL of 0.1 M HCl, then it was ultrasonicated for 30 min and transferred to an ice bath. Aniline monomer was added in a ratio of 5:1 and then oxidized with an appropriate amount of APS in 5 mL 0.1 M HCl and left in static conditions at −5°C for 36 h. The precipitate formed was filtered and then washed with distilled water and ethanol several times and then dried at 50 °C for 12 h in a vacuum oven. 10 µL of composite dispersion was dripped onto the surface of the GCE and then dried at room temperature. The synthesized PANI-Fe_2_O_3_-rGO/GCE sensor showed good selectivity, low detection limit, wide linear range, good stability and reproducibility, good anti-interference in detecting HQ. This electrode also facilitates rapid electron transfer between the electrode surface and the analyte, and effectively reduces the HQ diffusion length. The peak current produced by this electrode is strongly influenced by pH, therefore the electrolyte solution must be maintained at a certain pH.

In addition, there is a study by Mani et al. [107] which manufactures amperometric sensors for DA determination. This sensor is made of GCE modified with GNS nanocomposite and bismuth nanoparticles (BiNPs). GO prepared through the Hummers method [108] then the homogeneous dispersion was collected by ultrasonication for 2 h. Bi(NO_3_)_2_. 5H_2_O was added to 50 mL of GO dispersion (*w/w* 2:3) and ultrasonicated for 1 h. The mixture was added 0.5 mL of hydrazine monohydrate 32.1 mmol and refluxed at 160 °C for 24 h under a nitrogen atmosphere. The product was isolated and washed with plenty of water and ethanol after the reaction was complete. The obtained nanocomposites were dried overnight at 60 °C and redispersed in 0.5 mg/mL DMF. GCE to be modified is polished with alumina slurry and then cleaned and dried. 5 µL of the GNS-Bi nanocomposite dispersion was dropped onto the surface of the GCE and then dried at room temperature. The fabricated GNS-Bi/GCE electrodes exhibit excellent electrocatalytic capability against DA electrocatalysis. In addition, this sensor can detect DA sensitively and practically with a low detection limit and wide linear range, and has excellent stability, repeatability and reproducibility.

Meanwhile, in 2016, Yiǧit et al. [109] fabricated sensors for the simultaneous determination of PT, aspirin (ASA), and CAF. PT, ASA, and CAF are often combined in multidrug pharmaceutical formulations for the treatment of various types of tension pain, such as headaches, migraines, muscle aches, menstrual cramps, toothaches, arthritis, colds, or nasal congestion [110,111,112,113]. This sensor combines graphene and Nafion to modify the GCE. Graphene was synthesized by GO synthesis using the Hummers method [114] and continued with GO reduction using the irradiation procedure [115]. The graphene-Nafion composite film was prepared by adding graphene to a 0.5% *w/v* Nafion-alcohol solution and ultrasonicated to form a homogeneous solution. GCE was polished with alumina slurry on a microcloth polishing pad and rinsed with water until clean. GCE was sonicated successively in 1:1 nitric acid, ethanol, and water then rinsed with water and then dried at room temperature. Dispersion of the homogeneous solution of graphene-Nafion was dropped on GCE and dried at room temperature for 8 h. The electrodes were scanned cyclically with a potential of 0.2–1.65 V for 10 cycles at a speed of 100 mV/s. The proven fabricated graphene-Nafion/GCE sensor can be used for the simultaneous quantitative determination of PT, ASA, and CAF in pharmaceutical formulations due to its good anti-interference ability and selectivity. This sensor is quite sensitive, quite stable, and has satisfactory repeatability. The current generated by these electrodes is quite high during CV scanning, but the reduction peaks weaken when scanning is reversed.

Afkhami et al. [116] developed a sensor for the determination of omeprazole (OMZ) in real samples. OMZ is widely used as an antiulcer drug to control gastric acidity in patients with Zollinger-Ellison syndrome [117,118]. The manufacture of the Ni_0_._5_Zn_0_._5_Fe_2_O_4_/G/GCE sensor begins with the hydrothermal manufacture of Ni_0_._5_Zn_0_._5_Fe_2_O_4_/G nanocomposites. rGO was dispersed in H_2_O and stirred for 12 h to form a homogeneous dispersion and then added salt (Ni(NO_3_)_2_, Zn(NO_3_)_2_, and Fe(NO_3_)_3_) in an adequate and stoichiometric ratio into the rGO dispersion then stirred at room temperature. room. The pH of the solution was adjusted to 10 with the addition of NH_3_ slowly and then the solution was stored in a Teflon-coated autoclave at 180 °C for 24 h until crystallized. The resulting product was washed with deionized water several times and dried at 50 °C for 24 h. The GCE to be modified was polished and rinsed with distilled water and then sonicated in a 1:1 HNO_3_ solution, acetone, and double distilled water for 10 min each. GCE was activated in a scanning potential of −0.6–2.0 V in 1 mol/L H_2_SO_4_ solution at a rate of 100 mV/s. 1 mg G was dispersed in 5 mL of a mixture of DDW, ethanol, and sodium dodecyl sulphate (SDS) in a ratio of 3:1:1 *v/v* and sonicated for 30 min. 10 µL aliquot of the pre-prepared suspension was dripped directly on the prepared GCE surface and dried at room temperature to form a thin layer of graphene on the GCE surface. Next, the polished GCE was dropped with 10 µL of Ni_0_._5_Zn_0_._5_Fe_2_O_4_/G nanocomposite suspension and dried at room temperature. The prepared electrodes resulted in an increase in peak oxidation current and sensitivity, an increase in peak sharpness, and a decrease in the OMZ oxidation over-potential. In addition, the detection limit is low, the linear range is wide, stable, and the reproducibility is excellent. However, the high electroactivity of the electrodes can cause adsorption of OMZ oxidation products which can reduce the sensitivity of the sensor. Apetrei et al. [119] developed sensors to detect routines. Rutin is a flavonoid glycoside that is widely used as a pharmacologically active substance with antibacterial, antioxidant, tiviral, antitumor, and so on properties [120,121,122]. This sensor is made from a modified SPCE. The graphene dispersion was prepared by mixing 1 mg graphene and 1 mL of 0.2% CH solution in acetic acid pH 5.0. The graphene CH mixture was sonicated for 2 h to make a homogeneous dispersion. AuNPs were prepared by reduction of HAuCl_4_ with trisodium citrate. 1 mg AuNPs were added to the homogeneous dispersion of graphene-CH and ultrasonicated for 1 h. 10 µL of graphene-AuNPs composite dispersion was dropped on SPCE using a micropipette and then dried in a desiccator at room temperature. The prepared graphene-AuNPs/SPCE sensors can detect routines in pharmaceutical products with precision and accuracy, and can be prepared using a simple and low cost method.

Additionally, in 2020, Oghli & Soleymanpour [123] synthesized sensors for the determination of traces of paroxetine (PRX) in biological and pharmaceutical media. PRX is one of the drugs that is often used as an antidepressant which is generally used in high enough doses to optimize the effectiveness of therapy. [124]. The use of paroxetine in the long term has the potential to cause a serotonergic effect on the central nervous system which causes inhibition of neuronal serotonin reuptake [125]. Prior to modification, PGE was carefully polished on a smooth white paper and then coated with Teflon strip. Electrical connection to PGE is done with stainless steel wire. PGE is immersed vertically into the test solution. 5 mg of GO which has been synthesized using the Hummers method [108] was added to 5 mL of a solution containing 2 mM phosphotungistic acid (PWA) and 0.05 M PBS. The mixture was placed in an ultrasonic bath for 5 min to obtain a uniform suspension. The suspension was transferred to an electrochemical cell and deposited onto the PGE surface at a constant voltage of −1.2 V for 300 s. The synthesized rGO/PWA/PGE sensor can detect PRX sensitively and selectively with a low detection limit and a wide linear range. The high selectivity of this sensor allows the determination of PRX in a variety of real samples, such as tablets, human serum and urine samples. The stability and reproducibility of this sensor is not very high but still acceptable. The summary of graphene and GO utilization for pharmaceutical sensor is summarized in Table 3.

### 2.4. Nanodiamond

Diamond material has attracted numerous attention due to its high chemical and physical stability. Diamond has been doped and modified to give the catalytic activity. In 2021, Wang et al., developed a highly selective, reproducible, and stability monitoring of acetaminophen (APAP). Acetaminophen or paracetamol is a widely use drug as the most effective painkiller to relieve pain and fever. This sensor used boron doped diamond (BDD) as the electrode, due to its unique properties, such as large specific surface area, low background current, and fast electron transfer. Furthermore, this sensor can be applied in wide range application in electrochemical analysis of other drugs [126]. Various modified nanodiamond material for pharmaceutical sensor application is reported (Table 4).

In 2017, Simioni et al., fabricated a low cost, easy to prepare, and good stability electrochemical sensor using nanodiamond-DHP/GCE. This sensor form in thin film containing nanodiamond and DHP on GC electrode. The preparation of this electrode is by polishing the GCE to a mirror finish using an ultrafine sand paper and 1.0 and 0.5 μm alumina slurry. After being rinsed with ultrapure water, the polished GCE was sonicated for 2 min in isopropyl alcohol and ultrapure water, the dried in room temperature. The nanodiamond-DHP dispersion prepared by dispersed 1 mg nanodiamond and 1 mg DHP into 1 mL ultrapure water in ultrasonication bath for 30 min. modified GCE by nanodiamond-DHP dispersion by drop-casted 8 μL of to GCE polished surface and dried in room temperature for 2 h in air. By the modification of this electrode, it could improve the electrochemical performance by increasing the active area. This can be determined codeine (COD) in pharmaceutical and biological sample [127].

Recently in 2022, Wong et al., proposed nanodiamond/AuNP/PEDOT:PSS on scree-printed electrode (SPE) as electrochemical sensor for tryptophan detection [128]. Tryptophan is a medication used to treat patient with major depressive disorder (MDD), schizophrenia and bipolar disorder. Human body have a maximum dosage for tryptophan, to prevent unintentionally symptom due to overdose, we need to check tryptophan content in the body. One of which by electrochemical sensor. Nanodiamond/AuNP/PEDOT:PSS sensor could be a promising candidate due to its excellent selectivity toward tryptophan. The combination element in this sensor provide a fast, simple, and low-cost detection in non-invasive testing in food products [129]. The manufactured process to generate nanodiamond/AuNPs composite is by mixing 20 μL of PEDOT:PSS, 0.5 mg of nanodiamond and 250 mL of AuNPs were in 730 μL of ultrapure water, then, the mixture sonicated for 30 min to obtain a homogeneous stock dispersion containing a volume of 1.0 mL of nanodiamond-AuNPs. Applicability of this sensor can be used in determination tryptophan, dopamine, and caffeine in food sample.

In the work developed by Junior et al., 2021, they developed high surface area and high sensitivity of nanodiamond-MS/GCE sensor [129]. The incorporation on manioc starch in GCE allow high homogeneity and stability of the biofilm. The aim of this sensor development is for determine tetracycline in water sample (natural/tap water) and pharmaceuticals sample. Tetracycline (TC) is a well know antibiotics for their wide broad-spectrum of activity against both Gram-negative and Gram-positive bacteria [130]. The preparation of this sensor is by polishing GCE with activated alumina for 5 min on a piece of clean cotton fabric, and rinsed by ultrapure water. Then, 5.0 μL ND-MS dispersion dropped on the GCE surface, and the solvent was evaporated at 25 °C for 2 h. This sensor is sensitive and suitable for analysis and determination of TC in distribution water, and pharmaceuticals sample.

Other approaches have been utilized nanodiamond as electrochemical sensor in the work of Simioni et al., in 2017 [131]. In that work, they constructed electrochemical sensor by modifies glassy carbon electrode (GCE) with nanodiamond film. This sensor aimed to determine pyrazinamide (PZA) concentration in biological sample. Pyrazinamide is an antibiotic drug applied for treatment of tuberculosis. Overdose of this medication can generate side effect, such as nausea, vomiting, malaise, sideroblastic, etc. The manufacturer of this sensor started with polishing GCE with 0.05 μL alumina slurry until mirror finish and rinsed with ultrapure water followed by sonication using isopropyl alcohol for 2 min. Then the aliquot nanodiamond dispersion was dropped into GCE surface for about 8 μL. Lastly, the solvent was evaporated at room temperature. The combination between GCE and nanodiamond film can generate several advantages of the sensor, such as high electroactive surface area and faster electron transfer. Thus, could be improve the electrochemical activity of the sensor.

Nanodiamonds are also often used for sensors that can determine hormone level and neurochemical in the body. One of them is dopamine. Dopamine (DA) is an essential hormone and neurotransmitter in human body that correlated with movement, memory, emotional control, sleep, and attention. Upnormal number of DA is associated with neurological, equally Parkinson’s depression and Alzheimer [132]. Various approaches have been utilized to overcome this issue. For example, Chang et al., (2020) [133] develop electrochemical sensor using nitrogen-incorporated ovoid-shaped nanodiamond (NOND). The NOND film was synthesized using MPECVD method in pristine and patterned Si substrate. Then, the Si_3_N_4_ layer form during the initial stage following by the diffusion of nitrogen (N) atom and the NOND film was deposited. NOND have many excellent properties, such as polycrystalline grains with multiple orientations and amorphous carbon, thus the conductivity is enhanced as compared with the glassy carbon substrate. Moreover, NOND as sensor perform superior sensitivity, and outstanding sensitivity compared with the pristine NOND electrode. In this work also provide that NOND electrode raise redox peak twice higher that pristine electrode due to its high surface area of the ovoid shape ND, which exhibit high conductivity and electrochemical activity.

Another work related the determination of DA is proposed by Baccarin et al., in (2019) [132]. In this work, they proposed high sensitivity electrochemical based sensor using screen printed graphite microelectrodes (SPE) modified with nanodiamond. The use of SPE as substrate due to its low cost, reproducible, and disposable compared with traditional carbon electrode. The constructed of this SPE/nanodiamond sensor is by using drop casting method. On the other side, Puthongkham et al., also provide electrochemical sensor for determine DA using carbon microfiber electrode (CFME) modified with nanodiamond [134]. The usage of nanodiamond in this sensor is to increase the active site, sensitivity, and decrease fouling of the sensor. The manufacturer of this sensor is using drop casting method, where 25 μL nanodiamond dispersion was dropped to cover the CFME tip on a glass sline in a hot plate which accelerated solvent evaporation. nanodiamond/CFME was dried overnight at room temperature before use. There is COOH− functional particle in the electrode surface, which the size is affect the electrochemical properties. The nanodiamond/CFME exhibit electrocatalytic properties toward surface-sensitive redox species and low charge-transfer resistance. Not only as mentioned before, but this sensor also has high selectivity.

**Table 4:** Summary of nanodiamonds for pharmaceutical sensor application.

Electrode	Modifier	Detection Method	Target	Sample	LOD (μM)	Linear Range (μM)	Electrolyte	pH	% Recovery	Ref.
BDD	NG-BDD film	EIS, CV, DNPV	Acetaminophen	Tablet	0.005	0.02–50	PBS	7.4	98–103	[126]
GCE	ND-Graphite/chitosan	CV, SWV	Codeine	Human Serum, Urine	0.0545	0.299–10.8	PBS	5	87.8–100.8	[127]
Carbon-fiber microelectrodes (CFMEs)	ND-COOH	FSCV, EIS	Dopamine	Brain slice tissue	0.003	0–5.0	PBS	7.4	NA.	[134]
SPEs	ND-SPE	CV, DPV		Real samples	0.57	NA.	PBS	7.4		[132]
GCE	NOND	CV		Human Blood	0.054	0.1–100	PBS	7.2		[133]
					15	0.01–0.2				
					0.12	0.5–10				
SPEs	ND/AuNPs/PEDOT:PSS	SWV	Tryptophan	Milk, 70% Dark Chocolate, Synthetic urine	0.2	0.8–18	B-R Buffer (Britton Robinson)	4	95–103	[128]
GCE	ND/MS	CV, adtDPV	Tetracycline	Water sample	2:0 × 10−6 mol L^−1^	5.0 × 10−6 to 1.8 × 10−4 mol L^−1^	PBS	6.3	86–112	[129]

## 3. Drug Delivery Application

Nano-carbon material as a drug delivery, in simple terms, can be divided into two, namely carbon core and skin surface. In the skin surface, there is a targeting mechanism, detecting cancer cells among healthy cells, and binding cancer drugs that can release these drugs with certain supports, such as pH or temperature. Related reviews of nano-carbon material as a drug delivery in pharmaceutical applications intend to present comprehensive guideline for nanomedical researchers regarding the selection of nanocarbon as the application of the specific diseases they handle. Each different nanocarbon dimensions have the unique physical and chemical properties of each for pharmaceutical applications such as theranostics and, mostly, cancer therapy, which can be categorized into fullerenes and carbon-nanodot (C-dot) as 0D, CNT as 1D, graphene and GO as 2D, and graphite and nanodiamonds as 3D materials. The new mechanism is using magnetic metal. After being injected, the material is controlled by a magnet from the outside towards the tumor cells and there will be given heat control until the drug is released from the nanocarrier and the tumor cells are destroyed.

One of the factors considered in developing the role of semiconductor nanomaterials as nanocarrier for disease therapy is toxicity risk. A review examining the toxicological aspects of nanomaterials was discussed previously [135]. In 1986, Matsumura and Maeda explained the concept of targeting drugs to cancer cells by introducing the permeability-enhancing and retention (EPR) effect, an abnormality effect resulting from physical differences between cancerous tissue and healthy tissue, such as structural defects [136]. This property of EPR aids research in tumor targeting and accumulation of macromolecular drug carriers.

In addition, different nano sizes have been compared and found that the smaller the size, the greater the ability to penetrate into the cell nucleus but the greater DNA damage occurs [137]. Outperformed another semiconductor nanomaterial, nano-carbon has a high surface area, low toxicity, and biocompatibility becomes a promising drug delivery nanocarriers. The systematic role of nanocarbons in drugs for the controlled release and delivery of drugs or genes and their combination with targeted in vitro imaging was reviewed alongside the toxicological effects of using nanocarbons as theragnostic, recently, in 2019 [138]. The reports of no cytotoxicity or negligible cytotoxicity came from assays of nanocarbon composites on various types of cell models, e.g., fibroblast, epithelial, and endothelial cells by in vitro and mouse models, dog models, and chick chorioallantois membrane models by in vivo [139]. Complete while comparing the results of a carbon-based nanomaterial summary for drug delivery in vitro by Panwar et al. [138], this chapter will be focused on the discovery afterwards. The enthusiasm of nanomedical researchers made this field develop relatively fast and broad so it was appropriate to observe.

Density-functional theory (DFT) is a theory of calculating the electronic structure of atoms, molecules, and solids with a quantitative output, signals or pictures, based on the principles of quantum mechanics [140]. DFT has some limitations, one of them is the accuracy of the calculation of transition metal formation energy [141]. BIOVIA Materials Studio and Gaussian 09 or 16 are examples of software commonly used for DFT calculations. Many research reports included in this review prove their results based on DFT calculations, but we will not discuss DFT in detail because we focus on comparing the conclusions of each result.

### 3.1. Carbon Nanodots (C-Dots)

C-dots are 0D material below 10 nm in size and first reported by Xu et al. in 2004 [142] and then studied and divided according to their fluorescence into carbon quantum dots (CQD), carbon dots (CD), and C-dot by Sun et al. in 2006 [143]. Based on carbon precursors, conditions when synthesis, and its surfaces, C-dot is effectively prepared and utilized in the drug delivery application [144]. Nair et al. [145] shares a table of raw materials into various c-dot synthesis models, in buttom up and top down, and shows a comparison of the percentage of yields that each get. Further, Vale et al. [146] demonstrated the viability and safety of drug administration with three different c-dots from their own citric acid-urea synthesis; calcined-based, microwave-based, and hydrothermal-based, in popular normal breasts cell line model, MCF-10A, for 24, 48 and 72 h. Hydrothermal-based c-dot became the best drug delivery agent up to 72 h of treatment, as the lowest toxicity, evidenced by its significant viability profile and safety profile very close to antineoplastic non-tumor cells.

Rajendran et al. [147] opens a new path for the in-vivo therapy application in pharmaceuticals with mitochondrial detection in cancer cells from the Triphylyphosphonium (TPP) residue on the surface of C-dot. C-dot is synthesized from a mixture of TPP in distilled water and one amine precursor (PEG-di-NH_2_) which is heated at 140 °C at a certain time. The defined beat, centrifuged at 10,000 rpm for 10 min, and purified. This experiment produces a high green fluorescence quantum of 61% and could detect pharmaceutical drug, tetracycle, selectively and instantly based on real sample analysis they did.

One of the innovations of c-dot is magnetic natural carbon dot (MNCD), metal doped-c-dot which can be obtained by hydrothermal, coprecipitation, or carbonization via heating, which is famous for modern biomaterials such as drug delivery [148]. The magnetic properties of carbon dots mainly depend on the doping of magnetic metal ions or metal oxides, the famous one is Fe_3_O_4_/c-dot [149]. In theranostic applications, Gd^3+^/c-dot doping is used for tumor radiotherapy [150]. A study administered a tail vein injection and showed changes occurred over time when treated with Gd3+/c-dot, due to differences in signal quantity in each organ. Gd^3+^/c-dot accumulates in the kidneys and radiosensitivity increases markedly, indicating that it aids bladder cleansing. In addition, MNCD consist Mn, N, and S nanoenzymes incorporating c-dot can catalyze hydrogen peroxide (H_2_O_2_) to produce oxygen in solid-hypoxic tumors and is an option for photodynamic therapy (PDT) [151]. In vivo studies were carried out twice on zebrafish embryo. First, MNCD shows a high photoluminescence quantum yield of 17.7%. Then, the addition of hyaluronic acid (HA) to MNCD was carried out as a form of expansion of the application. HA pre-amina-functional will bind amine with active carboxylic acid group on the MNCD surface. MNCD/HA and doxorubicin (DOX), an anthracycline antitumor antibiotic used in chemotherapy [152] for several types of cancers of the lung, breast and ovaries [153], resulted in efficient detection of mouse melanoma (B16F1) cancer cells overexpression by CD44. This second study was then tested in vitro with confocal fluorescence microscope and MRI and confirmed the cytotoxicity of cancer cells B16F1 3: 2 of free doxorubicin treatment. MNCD/HA/DOX provides a much higher effect of chemotherapy against B16F1 with systematics and opens great cancer therapy opportunities related to c-dot/nanoenzyme engineering.

One alternative solution to obtain materials that are biocompatible and non-toxic to the human body is to synthesize materials by biosynthesis. Still with DOX carriers, carbon points can also be synthesized from bamboo leaf cellulose [154] and persimmon fruit [155] so as to reduce organic waste. Cellulose from the lignin content of bamboo leaves was extracted with the help of NaOH and NaOCl solutions for 2 h so as not to interfere with c-dot synthesis [154]. Cellulose is then heated to 250 °C so that carbonation occurs (combination of polysaccharides) to form a graphene oxide (GO) structure. Meanwhile, incomplete carbonation will form a c-dot which was confirmed by XRD at 2Ɵ 27.7 °C-dot synthesized from bamboo leaf cellulose was modified with 4-carboxybenzylboronic acid (CBBA) so that its specificity increased. In-vitro assays via confocal microscopy, blue fluorescence, reveal a cellular pathway to human cervical tumor cells (HeLa), used as treatment targets, via folate receptor-mediated endocytosis. Further, persimmon fruit is used as a carbon source in the synthesis of c-dot by non-solvent hydrothermal method which will be doped with nitrogen [155]. Persimmon c-dot (PCD) has many polar functional groups, hydroxyl (−OH), amine (−NH2), and carboxylic acid (-COOH), so it is very soluble in water and is able to bind anticancer drugs, DOX and gemcitabine (GEM) on the PCD surface via 1-ethyl-3-(3-dimethylaminopropyl) carbodiimide coupling reaction (EDC) coupling reaction. The presence of surface carboxylic acids and phenolic groups made both effective in inhibiting the proliferation of HeLa cells.

### 3.2. Fullerene

Fullerenes is another 0D carbon material. Some of the reasons why fullerenes and their derivatives [156] are good candidates for drug delivery and therapeutics are their immediate bioactivity when the surface is functionalized [157], can generate heat [158], pH-sensitive [159], and can selectively bind to microbial cells [160]. The use of fullerenes as drug carriers is the most widely used in cancer or tumor therapy, especially metal-doped-fullerenes. The use of fullerenes as drug carriers is the most widely used in cancer or tumor therapy [161], especially metal-doped-fullerenes. In recent years, the new discoveries have grown so much that they are interesting and deserve to be put together.

The fullerenes antiviral activity was based on the molecular structure and antioxidant activity of each type [162]. Recently, the new interaction studied was between the C_60_ fullerene and C_55_-containing transition metal N4 clusters (TMN4), Fe, Co, and Ni, which can bind to the nonsteroidal anti-inflammatory drug, ibuprofen (Ibp), as potential carriers for Ibp drug delivery [163]. The four doping structures are stable with different interaction distances, the longest at Ibp/C_60_ which indicates the possibility of physisorption while chemosorption by TMN4 with the shortest distance at Ibp/NiN4C55. The absorption energies of Ibp/C60, Ibp/FeN4C55, Ibp/CoN4C55, dan Ibp/NiN4C55 were respectively 13.14, 18.07, 17.81, and 23.11 kcal/mol, proving that fullerene TMN4 can be better for Ibp drug adsorption than pure C60. All these descriptions indicate that Ibp/NiN4C55 is the best, so the researchers then studied its reaction in an acidic pH environment, similar as target cell pH. The more acidic, the distance between NiN4C55 and Ibp is getting further and weaker until it is finally released. Unfortunately, this research does not show the fluorescence and the cell model that is used as a pH benchmark so that the track that Ibp/NiN4C55 passes is not very clear.

At the end of 2019, Christy et al. [164] reported the synthesis of magnetic sulfonated fullerene with a solvent role of diethyl ether and explained some of the profit reasons for using it as a drug carrier. Fullerenes and H_2_SO_4_ were mixed on ice then stirred for 3 h at 100 °C in an ultrasonic bath. This stirring aims to include the carboxylic acid group on the surface of the fullerenes. Then, an addition of water (50 °C) was carried out repeatedly, thus the sulfuric acid disappeared. Diethyl ether was added before the solution was filtered and dried. The synthesis process with the reaction that occurs during the synthesis is 2H_2_SO_4_ ↔ SO_3_ + HSO_4_ + H_3_O^+^, where C_60_ (fullerene derivative) + SO_3_ → C_60_ + SO_3_H and HSO_4_ + H_2_O → H_2_SO_4_ + H_2_O. Overall, this method is quite easy and efficient because fullerene oxidation is controlled to maintain the integrity of the basic spherical structure with C_60_SO_3_H synthesis results confirmed on FTIR and FT-Raman characterizations that image molecular symmetry. Not only UV–Vis and FT-RAMAN, FT-IR, XRD, SEM, HRTEM, XPS, EPR and VSM analyzes were also described, which confirmed the presence of Ag mode and superparamagnetic properties which can be controlled by an external magnetic field towards the target cell [165]. This finding definitely deserves further study for drug delivery applications.

The sensitivity of C_60_ fullerenes to pH functioned with carboxyl groups and the addition of trimethyl chitosan (TMC) polymer was tested with Molecular Dynamics (MD) simulations to study its potential as a carrier for cancer drugs, DOX and Paclitaxel (PAX) [159], a complementary drug to chemotherapy drugs as an increase in efficacy and reduce toxicity [166]. The experiment in neutral (drug absorption) and acidic (release) pH was carried out three times. First, by simply combining C60 with DOX or PAX, hydrogen bonds occur at neutral pH which trigger strong absorption between the carboxyl group of the C_60_ which has a negative surface charge and DOX which includes the amine group with a positive surface charge. This is not the case with PAX. Furthermore, when TMC was added, the van der Waals interaction and hydrogen bonding increased so that it could absorb both DOX and PAX. Finally, the combine of two (TMC-C_60_) showed a stable system on DOX, while PAX showed slower release so ultimately reduce the total amount of drug. Boron nitride (BN) is also chemically stable and non-cytotoxic in the human body when bound to C60 has also been studied as a carrier for hydroxyurea (HU) [167], an antiretroviral drug of a number of myeloproliferative, neoplastic, non-haematological diseases, and recently, Human Immunodeficiency Virus (HIV) [168] and commonly used in chemotherapy cancer [169]. HU is chemically adsorbed on the local pyramidal sites of BNFs by the carbonyl group on the C_60_ surface, which is optimal on the B36N36. The release process is similar to TMC-C_60_, the bond is weaker when the drug is under acidic conditions.

Metal-doped-Fullerene get enhances the electronic, magnetic, and adsorption energy [170]. The frequently reported parameter is using DFT. For example, in a research report fullerene Boron (B) which is covalently bound to Fullerene C_59_ as a cyclophamide (CTX) drug carrier, an immunosuppressive drug and effective therapy for many diseases, such as cancer and lupus [171,172,173]. From the various samples made, the covalent bond through the phosphoryl oxygen atom on the surface of B-C_59_ was investigated by DFT calculations to find out one sample is more stable and has more potential than other samples [174]. Also last year, the DFT calculation applied in the Gaussian program was also used to calculate the CNT-delivered intermediate cyclophosphamide (CPP) [175].

The performance of B, Al, and Si was tested as the carrier of HU, but this time compared to the pure-C_70_ [28]. In contrast to HU in pure-C_70_ which physisorption with charge transfer and absorption energy is very low to negligible, they found the absorption of HU chemically strong and stable in BC_69_, AlC_69_, and SiC_69_ with adsorption energies respectively 53.86, 84.46, and 71.13 kcal/mol, with the same release process as common drug delivery, the attack of H+ ions due to changes in environment pH from neutral to acidic. Other report, Nattagh et al. [47] demonstrated that B and N were stably inserted in C60 due to the negative energies of the formation of BC_59_, NC_59_, and BNC_58_ fullerenes. Anti-inflammatory and antiplatelet drugs which child-friendly [48] and usually used in forensic applications [176], Acetylsalicylic acid (Aspirin), which was initially weakly adsorbed on pure-C60 became stronger with adsorption energies of BC_59_, NC_59_, and BNC_58_ respectively 1.04, 0.41 and 0.96 eV. This strength adsorption due to the B-O covalent bond formed between Aspirin and BC_59_ or BNC_58_ while pure-C_60_ and NC_59_ are electrostatic.

Last year, Esrafili et al. [177], for the first time, reported the comparison of fullerene C_60_ with the alkali metals, Li, Na, and K, for delivery of 5-fluorouracil (5FU), an anticancer drug requiring intravenous administration, which is adsorbed on the surface of the material with adsorption energies, respectively, of 9.33, 16.58, and 14.07 kcal/mol. Realize the potential of alkali metals, pure-C_20_ fullerenes and (Calcium) Ca-decorated were reported for carrier sulfasalazine (SSZ), curcumin (CUR), and naproxen (NPX) adsorbed on their surfaces [178]. Materials were evaluated in a solvent (water) environment with functional CAM-B3LYP, a new hybrid exchange–correlation functional [179]. The CUR/Ca-C_20_ complex bond is stronger because it is through covalent interactions, while the SSZ//Ca-C_20_ and NPX//Ca-C_20_ complexes are through electrostatic interactions. The target of this therapy is the molecular docking process that initiates the inhibition of proinflammatory cytokines such as cyclooxygenase-2 (COX-2), tumor necrosis factor alpha (TNF-a) and interleukin-1b (IL-1b) that reduce cardiovascular risk in rheumatoid arthritis.

COVID-19 pandemic provoked new issues in nanocarriers research. One of COVID-19 drug is Chloroquine (CQ). Fullerene pure-C_60_ and C_59_-doped B, Al, and Si, some metal [180] with relative characteristics to carbon more than other metal, proved by DFT calculation, are one of the attractive potential CQ nanocarriers. The adsorption energy enhances from −4.1 kcal/mol Pure-C_60_ to the range −12 to −45 kcal/mol [170]. The drug properties of CQ did not change with this nanocarrier making it a worthy candidate for further testing.

### 3.3. CNTs

As a 1D material, the feasibility of CNT as drug carriers is focused on the synthesis, purification and, especially, chemical functionalization because the molecular composition of CNT is highly hydrophobic which makes it susceptible to aggregation and the material is almost insoluble in any solvent. The chemical functionalization strategy for integrating CNT into the biological environment most commonly uses the intravenous injection route, suitable for drug delivery to the kidney and urethra, and subcutaneous administration of CNT. The advantages and disadvantages of each path have been summarized previously [181,182].

The possibility of using macromolecular delivery vehicles to target small molecule drugs to tumor sites [136], CNTs, subdivided into Multi-Walled Carbon Nanotubes (MWCNTs) and Single Wall Carbon Nanotubes (SWCNTs), and, the newest, Stacked-Cup Carbon Nanotubes (SCCNTs) or Carbon Nanotube-Fibers (CNTFs) [183], are emerging as promising candidates [184,185] with various surface functionalization being developed by researchers.

One that is familiar is on DOX targeting. Targeting DOX to tumors, SWNT fragments can enter cells via endocytosis process [186]. Last year, for the first time hydroxyl and carboxyl functionalized SWCNTs were investigated based on DFT to optimize hydrogen bonding with DOX molecules using the method of atomic in molecular quantum theory (QTAIMs) using Gaussian 09 software package [62]. DOX and the outer surface of CNTs containing carbon atoms saturated by hydrogen or some other chemical fragments were functionalized to trigger the appearance of −COOH and −OH in stable water and gas phases, indicating the bond between drug molecules before and after adsorption to the outer surface of the CNT did not change significantly. Karimzadeh et al. [187] simulated DOX brought by SWCNT to determine the effect of alcohol consumption on its delivery efficiency. This is the first molecular dynamic simulation to evaluate the effect of the presence of ethanol which has shown that the possibility of DOX encapsulation in the SWCNT cavity decreases significantly which is presumably due to a decrease in Van der Waals interactions between DOX and SWCNTs. In addition, DOX/SWCNT takes longer to penetrate cell membranes.

For the first time, acid-treated MWCNTs (ox-CNTs) can interact electrostatically, hydrogen bonds, and van der Waals attractions, with guanidylated hyperbranched polyethyleneimine derivatives (GPEI25K and GPEI5K) so that the functionalization of oxCNT/GPEI successfully loads DOX with encapsulation efficiency up to 99.5% [188]. This nanocarrier system is based on a pH trigger. This system has been subjected to in vitro experiments on cancer and normal cells showing more efficient DOX loading and release at oxCNTs@GPEI25K than oxCNTs@GPEI5K.

Many modifications of CNTs have also been found in drug delivery applications with biomolecules, such as DNA and biopolymers [189]. CNT as DOX carrier functionalized with cytosine-rich ssDNA fragment [190]. Cytosine serves as a platform for storage and controlled DOX release based on a neutral to acidic pH change, which was described previously. At neutral pH, the alternating ssDNA/DOX encapsulated on the CNT blocks other DOX, forming random coils. When the pH changes to acid, the ssDNA fragment folds into an i-motif accompanied by the strength of the d-DOX interaction weakening and releasing. The presence of a carboxyl group due to the functionalization of CNTs allows covalent bonding with the DNA strand. N-ethyl-N-(3-dimethylaminopropyl) carbodiimide hydrochloride (EDC) was used as the linking agent.

SWCNT has also been functionalized with trimethyl chitosan (TMC) for DOX and PAX carriers [191]. The presence of TMC will increase the hydrophilicity and biocompatibility of the carrier, and has a positive surface charge at the same acidic pH as the amine group in DOX, so DOX will be released slowly at acidic pH. TMC also promotes the formation of additional hydrogen bonds in both drugs with SWCNT. The electrostatic energy of DOX-SWCNT is around 6000 kJ/mol and PAX-SWCNTs are almost zero with weak van der Waals interactions, making it promising as a drug carrier system. There is also an example of Functionalization of SWCNT with a copolymer, dimethyl acrylamide (DMAA)-TMC, which has also been investigated as a dual responsive conductor of DOX and PAX which is sensitive to pH and temperature [192]. These intelligent carrier nanohybrids are stable, selective, DFT approved, and sensitive to health and cancer cell.

Other cancer drugs, cyclophosphamide (CPP) [175] and methotrexate (MTX) [192] and are included in the, interpolymer complexes, poly(acrylic acid)/poly(ethylene glycol) (PAA/PEG)/MWCNT. MWCNT, obtained from chemical oxidation [193], binds PAA/PEG through a vacuum process and then binds the drug under phosphate buffer in ultrasonic conditions. Foci of release of both drugs kinetically at pH 7.4, 4 febrile temperature were also reported.

The application of CNTs as nanocarriers of the COVID-19 vaccine has been extensively studied. Another COVID-19 control drug under development is Remdesivir (RDV) which interacts with CNTs activated by the carboxylate group [194]. The CNT/RDV interaction was investigated by the B3LYP/3-21G method [195] and DFT calculation. CNTs have also been functionalized with Al, Si, and SiC in the molecular adsorption of Hydroxychloroquine (HCQ), a COVID-19 drug. Based on DFT calculations, Al-CNT is the best candidate for HCQ drug carrier because it has an absorption energy of −45.07 to −93.53 kcal/mol in the gas phase, and −43.02 to −88.97 kcal/mol in the liquid phase [196].

### 3.4. Graphene

Graphene is a 2D allotrope of carbon that is hexagonally arranged, sp^2^-bonded, nonmagnetic, and stable. Graphene is claimed to be one of the strongest materials because it has a tensile strength of 130.5 GPa and a Young’s modulus of 1 TPa [197]. The graphene derivatives such as graphene oxide, porous graphene/graphene oxide (GO), reduced graphene oxide, and graphene quantum dots, having mostly magnetic properties and sp^3^-bonded, is also a recent trend in nano-drug carriers. The advantages of each derivative and each method of graphene synthesis have been previously reported [198].

Graphene and GO have been reviewed in comparison as carriers of the anticancer drug, DOX, based on DFT calculations and fluorescence spectroscopy which confirmed the binding of DOX molecules was stronger in the sp^2^ than in the sp^3^ region [199]. The DOX bonds to sp^2^ and sp^3^ are stabilized by different bonds, the -π and CH-π interactions on sp^2^ Graphene and hydrogen bonds and -π interactions on sp^3^ GO. The different adsorption characteristics make Graphene and GO interesting for further research in drug delivery. Moreover, putting aside the weak DOX interaction in the sp^3^ region, GO has a nanostructure that has the potential to reduce DOX cytotoxicity caused by drug resistance and poor internalization [200]. This year, a study emerged revealing that the previously stated weakness of DOX absorption can be controlled by adding a hydroxyl group to GO [201]. The more hydroxyl groups, the stronger the electron affinity for DOX, further increasing the DOX loading capacity of GO. The order of absorption energy of the reported derivatives is GO-OH-DOX > GO-OH-O-DOX > GO-O-DOX.

The next promising graphene innovation is with polymer. GO was modified with amino silane functionalized Fe_3_O_4_-NH_2_ nanosheets. The –NH_2_ group of this functionalized GO nanosheets can react with the –COOH group of S-1-dodecyl-S′-(α,α′-dimethyl-α″-acetic acid)trithiocarbonate (DDMAT), one of the Reversible Addition-Fragmentation Chain Transfer Technique (RAFT) agents, a method for grafting polyglycidyl methacrylate (PGMA) [195,202]. After that, the PGMA chain epoxy ring was opened with a hydrazine group (N_2_H_4_) and binding DOX [203]. This synthesis is quite complex but exhibits pH sensitive behavior and excellent biocompatibility when tested on HeLa cells. The higher the incubation time of the cells, the higher the DOX red fluorescence intensity. Other polymers functionalized with GrO for binding DOX are some model poly(ethylene glycol) (PEG); common PEG [204], short-chain PEG consisting of 15 repeat units (Sh-PEGGO), and long-chain PEG consisting of 30 repeat units (L-PEGGO), were calculated by DFT calculations [205]. The longer the PEG chain, the stronger the DOX absorbed in PEG/GO, mediated by van der Waals forces (vdW), electrostatics, and the total interaction energy of GO−DOX and PEG−DOX contact areas. The existence of DOX aggregation is based on GO−DOX and PEG−DOX contact areas themselves. PEG was also investigated for binding to hydrazone bond (hy-PEG) and then mediated by folic acid (FA) ligand in tumor cell therapy with DOX. This 150 nm system is sensitive to pH and has been internalized via clathrindependent endocytosis and macropinocytosis in HeLa cells, and was shown to have higher cytotoxicity in GO/DOX-hyd-PEG-FA than GO/DOX-hyd-PEG [206].

PEG/GO measuring 146.1 nm was also developed as a cisplatin (pt) carrier, an anticancer chemotherapeutic agent for tumor hypoxia, a disorder in which the oxygen concentration is below tissue requirements [158], that contains and will form a platinum complex when it binds to DNA, causing the DNA chain to crosslink and then trigger cell death [207], with the help of H_2_O_2_ and succinic anhydride [204]. (PEG/GO)pt showed DOX absorption peaks at 232, 253, 291 and 480 nm based on UV analysis and is a dual drug delivery system worthy of further testing. The absorption peak (PEG/GO)pt-DOX was between the spectra of (PEG/GO)pt and DOX itself based on UV-Vis analysis and was released at pH 5.3 and 7.4, indicating a potential dual drug delivery system worthy of further testing.

Graphene was also successfully decorated with Chi to be a carrier for DOX and PAX. Chi-decorated GO will be a natural cationic polysaccharide to improve compatibility [208]. The absorb and release of DOX which can be controlled by Chi-decorated graphene by adjusting the pH of the solution to the protonation state of chitosan and the concentration and aggregation of DOX and chitosan molecules [209]. DOX molecules can bind to bare graphene surface that is not attached to Chi and also be adsorbed on top of the chitosan chain. Electrostatic and Van der Waals interactions are reported to be weak between DOX and the carrier, which also proves this system is very advantageous for cancer therapy [210]. The positive surface charge of TMC at acidic pH is the same as that of the amine group in DOX, which can facilitate drug release under acidic conditions. In the release of DOX, high concentrations of Chi and DOX will decrease the surface area of bare graphene and becomes conducive to DOX release based on the pH setting of the Chi protonation state. Next year, Farahani et al. [211] modified Graphene-Chi with Bovine Serum Albumin (BSA) which helps stabilize graphene-Chi and bind DOX. BSA(Graphene) nanocomposites in three different presentations, 0.5%, 2%, and 5% by weight of BSA, were dispersed in distilled water at pH 10 and then magnetically stirred with Chi-DOX. After all three samples showed low toxicity, they compared the DOX release response in vitro with in vivo and obtained consistent results in both, which was most optimal in the sample of 2% BSA(Graphene) established in SKBR-3 cells in 2D and 3D culture models at pH 4, 5 and 7.4 for 48 h.

Graphene and Graphene-TMC as a carrier system has bound PAX at blood pH by weak electrostatic and Van der Waals interactions and released PAX slowly so that it is advantageous for cancer therapy [210]. This system is sensitive to neutral and acidic pH. At neutral pH, there was no hydrogen bond between PAX and both systems, while at acidic pH it was present but not visible during characterization because the functionalized graphene had no surface charge. In terms of bonding, Graphene-TMC is a more stable PAX carrier than DOX with the same carrier.

Graphene has been modified to deliver the drug dexamethasone (DXM), which is popular for the treatment of COVID-19. More focused on surface morphology, Islam et al. [212] made DXM-nGO composites with antisolvent precipitation method with GO concentrations of 0.5 and 1% in solution. The increase in water solubility promotes an increase in the absorption and bioavailability of DXM in GO by up to 10 times.

### 3.5. Nanodiamonds

Nanodiamond is an around 5–50 nm 3D allotrope of carbon that is often used in biomedical and pharmaceutical applications because of its negligible toxicity, inert core, rich chemistry of the surface, and the bright and strong fluorescence [213]. Nanodiamond has been functionalized with Chi and Polypropylene (PP) mesh for hernia treatment and pelvic floor construction as an alternative to surgery with potential for blood clots, impaired renal function, and neurological disorders due to poor biocompatibility of conventional PP mesh [214]. Carboxylated nanodiamonds (cNDs) have also been reported to inhibit tumor cell migration [215]. cND prevent the epithelial-mesenchymal transition (EMT) process through the growth factor signaling pathway (TGF-β) thereby inhibiting Hela cell migration. As a reference for the application of post-test nanodiamond in test animals, such as mice, the safety of nanodiamond in large mammals using the intravenous (IV) and hepatic portal vein (HPV) injection methods has also been previously reported [216].

The drugs that ND is looking for are still very minimal. Recent discoveries are mostly confined to DOX. Most recently, in 2022, surface functionalized anodiamond with polymers using the photocatalytic atom transfer radical polymerization (ATRP) method were used for intracellular delivery of DOX [217]. The 2-methacryloyloxyethyl phosphorylcholine (MPC) polymer is dispersed in water so that it contributes too poor water dispersibility while improving overall system performance. FeBr_3_ as a photoredox catalyst because it is cheap by maintaining an appropriate therapeutic effect based on cell viability and cell imaging results. The previous year, DOX treatment with a nanodiamond carrier was successfully applied in the treatment of triple-negative breast cancer (TNBC) malignant tumors in mice [95]. Nanodiamond is coated with polyglycerol via a pH-sensitive hydrazone bond as the way it is released. This system exhibits different intracellular distribution profiles of free DOX, one of which is endoplasmic reticulum stress without substantial DNA damage like DOX and does not induce a significant DNA damage response as does free DOX. It will not trigger the multidrug resistance (MDR) mechanism associated with doxorubicin. It is rare to find a system that also describes the response to MDR. MDR is a natural inflammatory response of tumor cells in the form of an intrinsic mechanism that actively removes drug molecules from intracellular targets causing a decrease in the therapeutic efficacy of DOX and several other unrelated drugs. MDR is common causes in chemotherapy and responsible for more than 90% of deaths in cancer patients [218].

Intravenous injection of pure detonation-synthesized nanodiamond has been promoted as a cytokine absorber in the treatment of cytokine release syndrome (CRS) in the treatment COVID-19 [219]. This system has been tested on mice and exhibit low toxicity, fast-acting cytokine-absorption, and good mice survival rates. The research opportunities for novel systems based on nanodiamond as a drug carrier are very widely open because they are the least expensive compared to other carbon-based materials.

In summary, it is a fact that binding the drug to the carbon nanomaterials has the potential to decrease toxicity. The summary of carbon-based material for drug delivery application is in Table 5. Any kind of functionality will help better. All DFT calculations show that the average formation of the drug delivery model system by nanoparticles can be achieved based on energy values and also easy surface preparation. The full potential for binding drugs by these nanoparticles also allows them to be allocated as water purifiers from chemical drugs that may be dissolved. An example of what has been reported is CNT which helps purify water because it can bind to three types of anticancer drugs, cyclophosphamide, ifosfamide and 5-fluorouracil [220]. This possibility is also the other way around.

## 4. Other Applications

Functionalized carbon nanomaterial has been shown to be biocompatible. The properties and characteristics that have been studied provide clear evidence that the functionalized nano carbon has prospects for application in the biomedical/pharmaceutical field. Nanocarbons can pass through membranes carrying therapeutic drugs into targeted cells. Moreover, several carbon nanomaterials have shown strong antioxidant properties and their use can reduce reactive oxygen species (ROS) induced injury and reduce ROS damage in biological systems. In addition, carbon nanomaterials have found great potentials in multiple aspects of biomedicine due to their excellent chemical and physical properties.

### 4.1. Antioxidant Agent

Excessive formation of reactive oxygen species (ROS) can cause oxidative stress, which causes cell damage that can lead to cell death [225]. Therefore, cells possess an antioxidant network to scavenge over-produced ROS. Most antioxidants have a limited capacity and are unlikely to be able to cope with the explosion of radicals and unstable products. Carbon nanomaterials such as fullerenes, single-walled and multi-walled carbon nanotubes exhibit excellent antioxidant properties and have been used in various medical applications. Antioxidant carbon nanoparticles scavenge reactive oxygen species (ROS) with higher efficacy than other dietary and endogenous antioxidants [226]. As reported by Wang et al., that the characteristics of pure C_60_ antioxidant showed a higher antioxidant effect than the natural antioxidant vitamin E in preventing lipid peroxidation [227]. This shows that the use of nanomaterials for antioxidants shows better results than natural antioxidants such as vitamin E. Dugan et al., also reported the use of fullerenol as a neuroprotective agent in cultured cortical cells exposed to excitotoxic injury and apoptosis. The results of this study showed that fullerenol is a water-soluble antioxidant compound and reduces excitotoxic neuronal death after short exposure to NMDA by 80%, and AMPA by 65%, or kainate by 50% [228]. Yolanda et al., studied the use of carbon nanotubes (CNTs) and graphene (GP) for antioxidant tomato seedlings. The results of this study showed an increase in tolerance to various types of biotic or abiotic stress and also no toxic effects from each dose of nano carbon used [229]. Based on the research reported, nano carbon has a high potential for antioxidant activity. Therefore, nano carbon can be an attractive material for antioxidant and medical therapy.

### 4.2. Tissue Engineering

Tissue engineering aims to replace damaged organ tissue and avoid organ transplants which are time-consuming, complicated procedures, by creating biological replacements [230]. Due to their outstanding mechanical properties carbon nanomaterials can be designed for tissue engineering construction which facilitates the fabrication of a biocompatible scaffold exactly resembling the original extracellular matrix for fabricating artificial tissues. Carbon nanomaterials are considered as physical analogues of ECM components (e.g., collagen fibers) because of their similar dimensions. Biomaterials have an important role in tissue engineering because of their effects that can influence cell-cell interactions and modify cells [231]. Carbon nanotubes have been reportedly shown to be excellent fillers for electroactive scaffolds relevant for a wide range of tissue engineering applications. Xinfeng et al., reported the fabrication of F-US-tube nanocomposites to evaluate porosity in the pore structure of scaffolds and marrow stromal cell cultures. The study shows that F-US-tube nanocomposites reinforced the scaffolds although some mechanical enhancements were not significant due to sample variations [232]. CNTs are non-biodegradable, have morphological similarities to the native extracellular matrix and their capacity to promote cell adhesion makes them suitable for scaffold production for tissue regeneration. Mazatenta et al., reported SWCNTs containing hippocampal cells are suitable materials for tissue engineering and can be directly involved in the stimulation of brain circuit activity. Functionally non-targeted MWCNTs promote the growth and proliferation of pancreatic cancer cells, showing new avenues for in vitro cancer analysis [233]. A patent on the use of CNTs for tissue engineering application was invented by Laurencin et al. CNTs was fabricated to be a composite scaffold and applied for bone repair and regeneration. This scaffolds is claimed to be biocompatible and biodegradable [234]. In other hand graphene and its derivatives have interesting characteristics as well such as high conductivity. As reported by adam et al., who made 3D printable graphene (3DG) composites with a higher conductivity level than other carbon composite materials. Ahmed et al., reported that biocompatible GO scaffolds promoted cell proliferation and HFB4 cell attachment to the fibrous composition [235]. The use of GO nanomaterials can not only improve the mechanical properties but can also adapt the nanofibrous scaffolds with suitable behavior for biomedical applications. Diamond nanoparticles also have excellent mechanical properties such as high surface area and good biocompatibility. Zhang et al., reported a study about nanodiamonds which were used as poly(L-lactic acid) and octadecylamine for use as bone scaffolds [236].

### 4.3. Bioimaging Application

Bioimaging is a noninvasive imaging technique to visualize living organisms, organs, cells, or biological activity over a period of time [237]. Bioimaging helps study the function of certain organs under different conditions in correlation with anatomy of the 3D sample. Carbon nanomaterials have the potential to be used as multimodal bioimaging of cells and tissues due to their excellent water solubility, biocompatibility and minimal cytotoxicity, as well as their outstanding tolerance to photobleaching. Tang et al., reported the fabrication of single-stranded DNA adsorbed on the surface of GO sheets effectively providing protection from enzymatic cleavage. SsDNA adsorbed to the GO surface is retained prior to enzymatic cleavage by a steric barrier effect that prevents DNase from binding to DNA for enzymatic digestion [238]. Hu et al., also reported the successful development of QD-rGO nanocomposite to function as an imaging agent in the visible region and a photothermal cancer therapeutic agent in the NIR region [239]. The thermal effect of irradiated rGO can not only kill cells, but also degrade QD which also serves as an optical indicator for heat dose and therapeutic treatment progress. The development of nanoscale is expected to open new avenues for radiation therapy in general in clinical applications. Study discussing carbon nanoparticles for bioimaging applications reported by Tan et al., mesoporous silica nanoparticles (MSN) coated with fluorescent fullerenes were successfully used as nanocarriers which demonstrated a pH-sensitive drug release capacity and could serve as fluorescent cell imaging. In vitro studies showed that the fabricated materials exhibited excellent biocompatibility, and the DOX-loaded nanocarriers exhibited efficient anti-cancer capabilities [240]. Meanwhile, a patent invented by Takeuchi claimed the SWCNT dispersion fluid for bioimaging. This material is claimed to be nontoxic and does not block the blood vessels in the lung when administrated into the organism [241].

## 5. Summary and Outlook

In this review the use of carbon nanomaterials has been discussed and shows that almost all carbon nanomaterials have potential to be applied in various fields, especially in the biomedical/pharmaceutical field. Various types of carbon nanomaterials or their derivatives which have outstanding structural and chemical properties have been discussed. These properties of carbon nanomaterials make them useful in a wide range of applications such as sensor application, gene or drug carriers, bioimaging, carriers in scaffolds for tissue engineering, each of which has been demonstrated using existing studies. First, carbon-based nanomaterial used as sensor application. Various carbon functionalized material is presented. The optical, electrical and chemical properties of carbon nanomaterials make them suitable for sensor applications and greatly affects the results of the validation methods. Outstanding features such as wide potential window, high conductivity, chemical and electrochemical inertness, low capacitance, biocompatibility, ease of modification, low cost, excellent sensitivity, and low detection limit make it widely used for biosensing applications in drugs or other materials. Second, carbon nanomaterial is used for drug delivery application. Previous studies have clearly demonstrated that significant and exciting advances in the characteristics of carbon nanomaterials such as their size adaptability, high surface area and capacity to bind drugs/biomolecules to enhance absorption across plasma membranes make them a good carrier medium for drug encapsulation and delivery to the plasma membrane in various parts of the body. Finally, not only sensor and drug delivery application, carbon nanomaterial has been used for other application in pharmaceutical area, such as antioxidant agent, tissue engineering, and bioimaging application. Existing studies explain that the use of carbon nanomaterials shows better antioxidant properties than natural antioxidants such as vitamin E [227]. Researchers have overcome the cytotoxicity of several types of carbon nanomaterials by making composites with more compatible materials. Thus, their use as nanocarriers for drugs and genes, as contrast agents for imaging, as scaffolds for tissue regeneration and various biomedical applications can be carried out with good results. This review shows the great properties of carbon nanomaterial for various pharmaceutical application that may provide point of view to develop a new carbon functionalized material in the future.

## Figures and Tables

**Table 2:** Summary of fullerene utilization for pharmaceutical electrochemical sensor application.

Electrode	Modifier	Detection Method	Target	Sample	LOD (µM)	Linear Range (µM)	Electrolyte	pH	Recovery (%)	Ref.
GCE	C_60_	CV, SWV, DPV	BPA	Wastewater	0.0037	0.074–0.23	KCl	8.0	94.0–104.0	[46]
	C_60_-CNT/IL	CV	NE	Serum, urine	0.018	0.07–30.0, 30.0–750.0	ACN	7.0	98.0	[50]
			IP		0.022	0.1–25.0, 25.0–700.0			101.0	
			DA		0.015	0.06–25.0, 25.0–800.0			98.5	
	C_60_-CNT/IL	CV, DPV	Hydrazine, hydroxylamine	Tap water	0.017	0.05–700.0	ACN	7.0	98.0–101.0	[51]
				Auxiliary cooling water	0.028	1.0–300.0			99.0–101.0	
	AC_60_-PdNPs	CV, DPV	DA	Pharmaceutical formulations	0.056	0.35–133.35	PBS	7.0	97.0–97.7	[54]
	GO-C_60_	CV, DPV	DA	Rat brain	0.008	0.02–73.5	PBS	7.0	101.8	[57]
				DA injection					94.0–100.16	
	C_60_-CNT	CV, DPV	L-dopa	Real samples	0.035	0.5–2000.0	PBS	7.0	99.8–100.4	[63]
			AC						99.25–100.16	
	C_60_-CNT/IL	CV, DPV	Diazepam	Urine	0.087	0.3–700.0	PBS	7.0	96.0–103.6	[67]
				Serum					96.3–102.8	
	NiNPs-CuNPs@reduced-C_60_	SWV	Vitamin D3	Serum	0.0025	1.25–475.0	KCl, LiClO_4_	7.0	98.6–100.5	[69]
				Urine					98.2–99.8	
				Tablet					96.8–98.1	
	Pt/C_60_	CV, DPV, EIS	CC	Local river water, sanitary wastewater	2.97	50.0–1500.0	PBS	7.0	96.2–103.8	[72]
			HQ		2.19	50.0–1100.0			95.7–104.4	
	C_60_/MWCNT/Nafion	CV, DPV, EIS	CAF	Brupanas	0.07289	10.0–1000.0	HClO_4_	1.0	97.71	[76]
				Pipadol Extra					95.66	
CPE	CuNPs/C_60_/MWCNTs	SWV	PT	Human blood serum, plasma, urine	0.000073	0.004–0.04	PBS	6.8	99.21–103.0	[60]
	MnO_2_/C_60_	CV, DPV, EIS	RFX	Pharmaceutical formulations	0.000968	0.001–0.04	BR buffer	3.0	98.98–101.52	[82]
SPE	C_60_-rGO-Nafion	SWV	MTZ	Synthetic serum and urine	0.21	0.25–34.0	PBS	7.0	92.0–100.0	[83]

**Table 3:** Summary of graphene utilization for pharmaceutical sensor.

Electrode	Modifier	Detection Method	Target	Sample	LOD (µM)	Linear Range (µM)	Electrolyte	pH	Recovery (%)	Ref.
GCE	GO	CV, LSV	MD	Serum, urine	0.1	0.3–200.0	PBS	7.4	95.6–104.3	[86]
	graphene	DPV	L-dopa	Tablet, mouse brain extract	0.022	0.04–79.0	PBS	6.2	101.4–109.42	[87]
	GNRs-GO-CNTP	SWV	Indomethacin	Human blood serum, urine, pharmaceutical	0.017	0.2–0.9, 2.5–91.5	PBS	8.0	98.0–103.5	[90]
	graphene/TiO_2_/PANI	SWV	ARP	Pharmaceutical formulations	0.0022	0.0112–0.0893	BR buffer	2.5	99.0–101.6	[100]
	PANI-Fe_2_O_3_-rGO	CV, LSV, DPV	HQ	Tap water	0.06	0.1–550.0	PBS	2.5	-	[104]
	graphene nanoparticles-Bi	CV, LSV	DA	Commercial injection	0.35	1.0–30.0	PBS	7.0	98.2–98.4	[107]
	graphene-Nafion	CV, SWAdASV	PT	Commercial tablet	0.0012	0.0083–0.51	PBS	2.5	-	[109]
			ASA		0.065	0.087–17.0				
			CAF		0.038	0.26–10.0				
	Ni_0.5_Zn_0.5_Fe_2_O_4_/graphene	CV, DPV	OMZ	Serum	0.015	0.03–100.0	PBS	6.0	97.5–101.1	[116]
				OMZ capsule					98.6	
GPE	graphene/2,7-BF	CV, SWV, CA	Methyldopa	Tablet	0.05	0.09–500.0	PBS	7.0	97.6–102.0	[93]
				Urine					98.7–103.2	
Pd	graphene	EIS, CV, SWV	NE	Human urine	0.06744	0.5–500.0	PBS	7.2	97.2–98.86	[94]
SPCE	graphene-AuNPs	CV, SWV	Rutin	Tablet	0.011	0.1–15.0	ABS	5.0	96.52–102.97	[119]
PGE	rGO/PWA	DPV	PRX	Tablet	0.0009	0.008–1.0	BR buffer	7.0	97.6	[123]
				Urine					98.4–101.6	
				Serum					97.7–101.3	

**Table 5:** Summary of carbon-based material for drug delivery application.

Carbon Materials	Incorporated Molecule	Drug	Usage	Ref
C-dot	C, N, and O	DOX	Evaluate the safety of three different carbon dots.	[146]
	TPP(PEG-di-NH_2_)		Anticancer	[147]
	Gd^3+^		Bladder cleansing.	[149]
	Mn, N, S, and HA		Anticancer, an option for PDT.	[151]
	CBBA		Anticancer	[154]
	EDC	DOX	Antitumor	[155]
		GEM		
Fullerene C_20_	Ca	SSZ	Reduce cardiovascular risk in rheumatoid arthritis	[178]
		CUR		
		NPX		
Fullerene C_55_	FeN_4_	Ibuprofen	Nonsteroidal anti-inflammatory drug	[163]
	CoN_4_			
	NiN_4_			
Fullerene C_58_	BN	Aspirin	Anti-inflammatory and antiplatelet drug	[221]
Fullerene C_59_	B	CQ	Treatment of COVID-19	[194]
	Al			
	Si			
	B	Aspirin	Anti-inflammatory and antiplatelet drug	[221]
	N			
	B	CTX	Anti-cancer	[174]
Fullerene C_60_	SO_3_H	Ibuprofen	Nonsteroidal anti-inflammatory drug	[163]
			Discovery of new superparamagnetic materials	[164]
	TMC	DOX	Anti-cancer	[159]
	TMC	PAX		
	B_36_N_36_	HU	Anti-cancer	[167]
		CQ	Treatment of COVID-19	[180]
		Aspirin	Anti-inflammatory and antiplatelet drug	[221]
	Li	5FU	Anti-cancer	[177]
	Na			
	K			
Fullerene C_69_	B	HU	Anti-cancer	[222]
	Al			
	Si			
Fullerene C_70_		HU	Anti-cancer	[222]
CNT	-	CPP	Anti-cancer	[175]
	-	RDV	Treatment of COVID-19	[194]
	S			
	Si			
	Al			
	COOH			
	-	CPP	Anti-cancer	[175]
	ssDNA	DOX	Anti-cancer	[190]
	Al-	HCQ	Treatment of COVID-19	[196]
	Si-			
	SiC-			
SWCNT	−COOH and −OH	DOX	Anti-cancer	[223]
	TMC	DOX	Anti-cancer	[210]
		PAX		
	DMAA-TMC	DOX	Anti-cancer	[191]
		PAX		
	Ethanol	DOX	Anti-cancer	[187]
MWCNT	PAA/PEG	CPP	Anti-cancer	[192]
		MTX		
oxCNTs	GPEI	DOX	Anti-cancer	[188]
Graphene	-	DOX	Anti-cancer	[199]
	Chi			[209]
	TMC			[210]
	BSA and Chi			[211]
	TMC	PAX		[210]
GO	-	DOX	Anti-cancer	[199]
	OH	DOX	Anti-cancer	[201]
	OH-O			
	O			
	PGMA	DOX	Anti-cancer	[202]
	PEG	DOX	Anti-cancer	[205]
	Sh-PEG			
	L-PEG			
	hy-PEG-FA	DOX	Anti-tumor	[206]
	hy-PEG			
	PEG	Pt	Anti-tumor hypoxia	[204]
		Pt-DOX	Dual drug anti-tumor hypoxia	
	-	DXM	Treatment of COVID-19	[212]
Nanodiamond	-	-	The safety of ND in large mammals	[216]
	MPC	DOX	Anti-tumor	[217]
	polyglycerol	DOX	Anti-cancer	[224]
	-	Cytokine	Treatment of COVID-19	[219]

## Data Availability

The data presented in this study are available on request from the corresponding author.
